# Unveiling the immunosuppressive landscape of pancreatic ductal adenocarcinoma: implications for innovative immunotherapy strategies

**DOI:** 10.3389/fonc.2024.1349308

**Published:** 2024-03-25

**Authors:** Songyu Guo, Zhenxia Wang

**Affiliations:** ^1^ First Clinical Medical College, Inner Mongolia Medical University, Hohhot, China; ^2^ Department of Hepatic-Biliary-Pancreatic Surgery, The Affiliated Hospital of Inner Mongolia Medical University, Hohhot, China

**Keywords:** pancreatic ductal adenocarcinoma (PDAC), tumor microenvironment, immunosuppression, immune cells, immunotherapy

## Abstract

Pancreatic cancer, particularly pancreatic ductal adenocarcinoma (PDAC), stands as the fourth leading cause of cancer-related deaths in the United States, marked by challenging treatment and dismal prognoses. As immunotherapy emerges as a promising avenue for mitigating PDAC’s malignant progression, a comprehensive understanding of the tumor’s immunosuppressive characteristics becomes imperative. This paper systematically delves into the intricate immunosuppressive network within PDAC, spotlighting the significant crosstalk between immunosuppressive cells and factors in the hypoxic acidic pancreatic tumor microenvironment. By elucidating these mechanisms, we aim to provide insights into potential immunotherapy strategies and treatment targets, laying the groundwork for future studies on PDAC immunosuppression. Recognizing the profound impact of immunosuppression on PDAC invasion and metastasis, this discussion aims to catalyze the development of more effective and targeted immunotherapies for PDAC patients.

## 1 Introduction

Pancreatic cancer is an extremely malignant digestive system disease. Because its gradually increasing incidence rate and mortality are very considerable in all diseases. A recent joint report by the following four agencies, including the American Cancer Society, the Centres for Disease Control and Prevention, the National Cancer Institute, and the North American Association of Central Cancer Registries, demonstrates that 3% of diagnosed cancer cases are pancreatic cancer patients, but they account for 8% of cancer deaths. Pancreatic cancer is in the top five causes of cancer death in the United States (1). Although the trend of incidence rate and mortality of pancreatic cancer is extremely changeable in the world, the discrepancy in the overall five-year survival rate between developed countries and developing is tiny, at about 6% (2).

Owing to its poor prognosis, it is regarded as a clinical disease in urgent need of breakthrough research achievements. Since pancreatic ductal adenocarcinoma (PDAC) accounts for approximately 90% of pancreatic tumors, the exploration and clinical practice of pancreatic cancer mostly focus on PDAC (3). One of the important reasons for the high mortality rate for pancreatic cancer is its characteristic of hiding early symptoms (4). About 50% of PDAC patients have reached locally advanced stages or have distant metastasis when they were diagnosed, which makes surgical treatment more difficult than for other digestive system tumors (5). Surgical treatment of PDAC is characterized by complex resection of the primary site and severe postoperative complications. The 5-year survival rate of patients undergoing surgery is less than 30% (6). Other effective methods for pancreatic cancer, including adjuvant chemotherapy, radiotherapy, targeted therapy, and immunotherapy, cannot benefit the majority of PDAC patients, and can only play an active role in specific subgroups of PDAC patients, which shows that further research on the treatment of PDAC is of great significance.

At present, although neoadjuvant chemotherapy drugs for pancreatic cancer have played a certain therapeutic role, the therapeutic effect is not as expected. It may lead to bone marrow suppression, resulting in tumor-promoting immune deficiency contrary to the therapeutic purpose (7). Immunotherapy offers several emerging technologies, including immune checkpoint block (ICB) through the use of immune checkpoint inhibitors (ICI), vaccines, and adoptive cell therapy (8). These treatment methods are currently in the realm of research and exploration due to numerous unresolved technical issues. With considerable effort, they can be widely applied in clinical settings. However, some treatments have been proven to be a reasonable choice and have positive impact on patients with special indications. For example, pembrolizumab is the first-line immunotherapy for PDAC, which has better efficacy for PDAC patients with the Microsatellite Instability-High/Deficient Mismatch Repair molecular subtype (MSI-H/dMMR). It is important to note that this subtype only accounts for 0-2.00% of PDAC patients (9). PDAC has a unique tumor microenvironment (TME) where various cells (mainly tumor cells, endothelial cells, immune cells, fibroblasts, nearby matrix, and microvessels) and substances (mainly cytokines, chemokines) interact to form a complex crosstalk network (10, 11). As the tumor progresses, some components of the TME may shift from inhibiting tumor growth to promoting it. Together, they form a composite microcosmic whole and have the characteristics of sustained immune inflammatory reactions, hypoxia, acidic environment, and high pressure (12). TME plays a crucial role in tumor growth, invasion, immune evasion, and drug resistance. Immunotherapy research is in full swing. Although immunotherapy research has made significant strides in treating melanoma and other tumors, its impact on PDAC treatment has been disappointing. Research on immunosuppression and immunotherapy of PDAC is still in the exploratory stage. We are required to gather more ideas and actions to promote immunotherapy to make most patients benefit.

## 2 Matrix of PDAC tumor microenvironment

TME of different PDAC individuals is highly heterogeneous. PDAC has been classified in many ways in the past. But for the study of TME, PDAC can be divided into Basal-like A/B, Classical A/B and Hybrids. Classical PDAC is more common in the early stage of disease. Basal-like PDAC is invasive. Basal-like A PDAC is associated with tumor metastasis. Basal-like B PDAC is associated with resectable diseases. Hybrid PDAC has the above characteristics. Recent studies have revealed that PDAC has both Basal-like and Classical types. With the progression of the disease, the intratumoral coexistence will be aggravated. This situation will lead to a worse prognosis of PDAC patients (13). TME plays an essential role in the immunosuppression of PDAC, inhibiting the penetration of immune cells, recruiting immunosuppressive cells, promoting immune escape, and contributing to the occurrence and development of tumors (14). The desmoplastic stroma accounts for 80% of the tumor volume in TME. The extensive matrix comprises stromal cells, extracellular components, and vascular structures. The stromal cells mainly have pancreatic stellate cells (PSCs), cancer-associated fibroblasts (CAFs), vascular-associated smooth muscle cells, and mesenchymal stem cells. The extracellular matrix, mainly composed of collagen, hyaluronic acid, and fibronectin, is jointly secreted by tumor cells and stromal cells (15). Fibroblasts can respond to carcinogenic signals even in the early stage of pancreatic lesions, including chronic pancreatitis. Fibrous tissue can be observed in the microstructure of low-grade precancerous pancreatic intraepithelial neoplasia (PanIN) (16). The surrounding matrix envelops pancreatic cancer, forming a solid tumor. Some studies have demonstrated that the microenvironment of metastatic foci also undergoes changes similar to those of the primary focus (17). The dense matrix acts as a physical and chemical barrier, inhibiting immune infiltration. This makes it difficult for the immune cells, neoadjuvant chemotherapy drugs, and immunotherapy to approach the cancer cells to play their due anti-cancer effects. This shows the abnormal function of local immune cells (18). The extracellular matrix (ECM) is a critical component that can promote tumor growth. Tumor cell components interact with ECM in complex ways. ECM can provide structural support and biomechanical signals for the growth of cancer cells (19). Additionally, ECM supports the formation and stability of blood vessels. Tumor cells and matrix cells release a variety of proteases, such as matrix metalloproteinases. Various angiogenesis-promoting substances, such as vascular endothelial growth factor (VEGF), fibroblast growth factor, and transforming growth factor, are hydrolyzed and activated by them, which are contained in ECM (20). Moreover, irregular fibrous in ECM tissue can promote tumor angiogenesis by encouraging the migration of immunosuppressive cells, such as CAFs and tumor-associated macrophages (TAMs) (21). The rich and dense fibrogenic matrix has a dual effect on tumor growth. On one hand, it promotes the formation of vascular structures, which can stimulate tumor blood metastasis. On the other hand, it can lead to vascular collapse and increased interstitial fluid pressure (IFP), creating an anoxic and low PH environment that is conducive to tumor growth (22). Under these conditions, TME prevents normal pancreatic cells and immune cells from accessing nutrients and drugs from reaching around tumor cells, leading to immunosuppression and drug resistance in PDAC (23). Due to the aforementioned factors, the PDAC stroma is considered a critical element that affects the beneficial impact of ICB. Several trials on anti-matrix therapy have been conducted, targeting transforming growth factor-β (TGF-β), focal adhesion kinase (FAK) signal transduction, and glutamine metabolism. These trials suggest that combining anti-matrix therapy with immunotherapy can ameliorate therapeutic efficacy (24). Matrix-targeted therapy is a potential approach to eliminate immunosuppression and alleviate the tumor-promoting TME (25). For example, some scholars have studied drugs that can target hyaluronic acid. PEGylated hyaluronidase (PEGPH20) refers to a polyethylene glycol nanocomposite composed of recombinant human hyaluronidase. PEGPH20 has been repeatedly proven to have the ability to degrade hyaluronidase and improve angiogenesis. PEGPH20 can also enhance the efficacy of chemotherapy drugs (25). TME is not a static entity and varies unevenly with tumor progression (26). The rich proteolytic enzymes in the ECM play a crucial role in stroma remodeling to accommodate the tumor heterogeneity of the matrix at different stages of tumor development (8). Additionally, the ECM contains integrins, a class of significant substances that serve as the primary receptor of adhesion molecules. It regulates cell movement and may contribute to the proliferation and metastasis of cancer cells, forming a metastasis niche. Its adaptive function is strong and it plays a role in various stages of cancer (27, 28).

TME is the key to the formation of metastasis niche. Researchers have reported a rare case of bone metastasis from PDAC (29). The variable incidence of the disease is 5-20%. PDAC cells are mainly colonized in the right iliac wing and D11 and L3 vertebral bodies. The advantages of osteoblastic or osteolytic changes have not yet been determined. The patient was treated with gemcitabine plus nab‐paclitaxel combined with zoledronic acid. Zoledronic acid can inhibit the growth of PDAC, stimulate anti-tumor response through γδ‐ Type T cells, and affect patient survival. The efficacy of zoledronic acid is also associated with TAMs.

The Pre-Metastatic Niche (PMN) plays an important role in the proliferation, colonization, and survival of tumor cells (30). Cytokines and chemokines, such as VEGFA, TGF-β, and TNF-α, are released by tumor cells, stromal cells, and immune cells, and they regulate PMNs of distant organs, making them suitable for the metastatic growth of cancer cells recruited (31). In the case of PDAC, PMNs not only assist in tumor metastasis but also promote fibrosis in liver metastasis sites (32). In addition, PMNs promote the recruitment of atypical immune cells derived from the bone marrow (31). The composition of infiltrating immune cells and the immunosuppression generated by complex crosstalk in TME can be determined by non-immune cells. Immune-mediated signals controlled by these cells can also regulate tumor invasion and metastasis.

Recent studies have demonstrated that the tumor matrix may have inhibitory effects on the development of PDAC, despite the general belief that it is tumor-promoting (33). In a word, the dense matrix components can have contradictory effects, promoting tumor cell apoptosis while also inducing tumor cell proliferation and migration. The type and location of the tumor may play a role in determining these effects.

## 3 Major cellular components in the TME of PDAC

**Figure 1:**
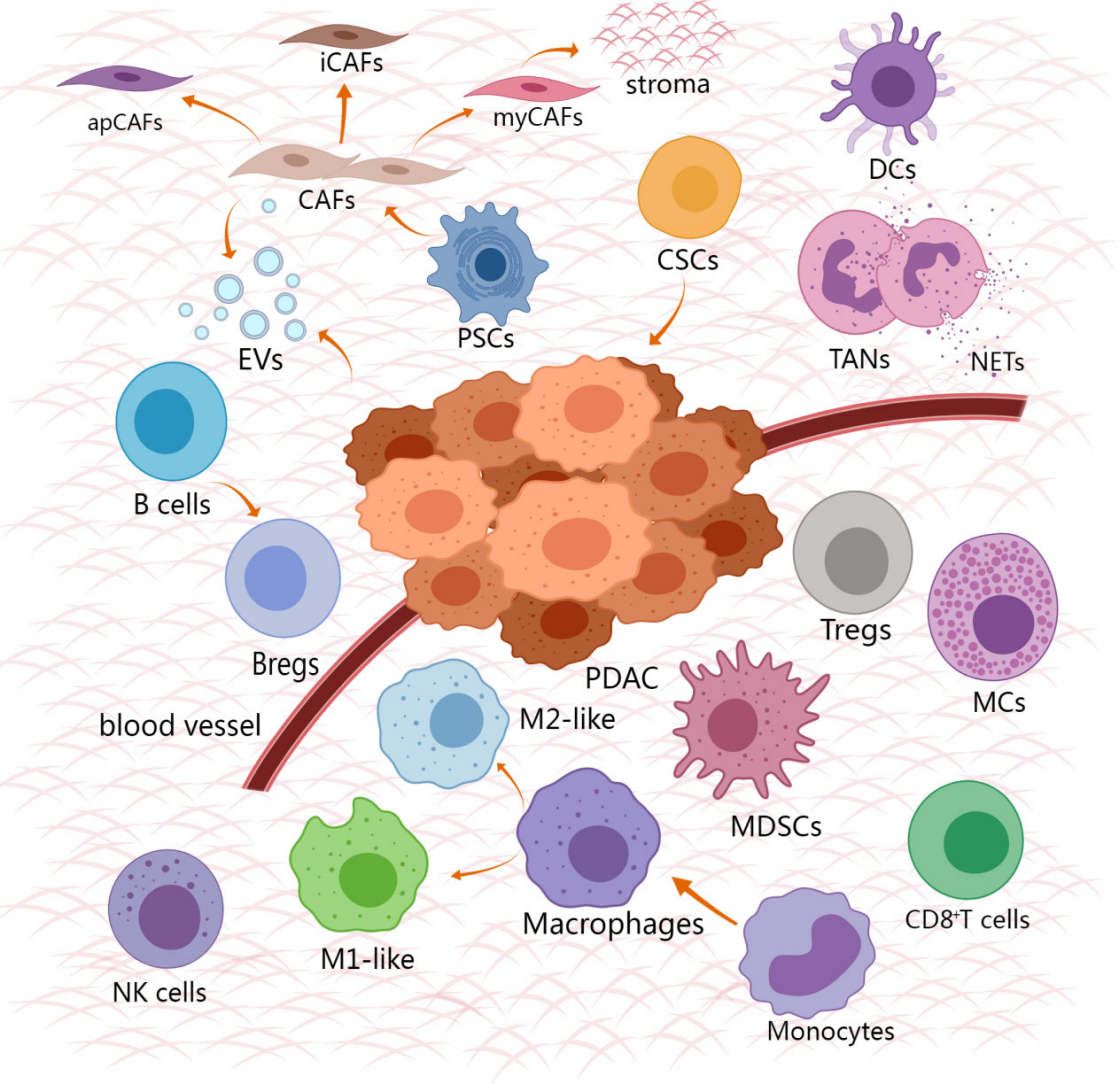
Cell composition and relative spatial distribution in TME of PDAC. The cell composition of TME is extremely complex, including tumor cells, stromal cells and immune cells. The immune cells that promote the tumor surround the tumor, and the anti-tumor immune cells are excluded from the distant tumor. Created with MedPeer (www.medpeer.cn).

**Figure 2:**
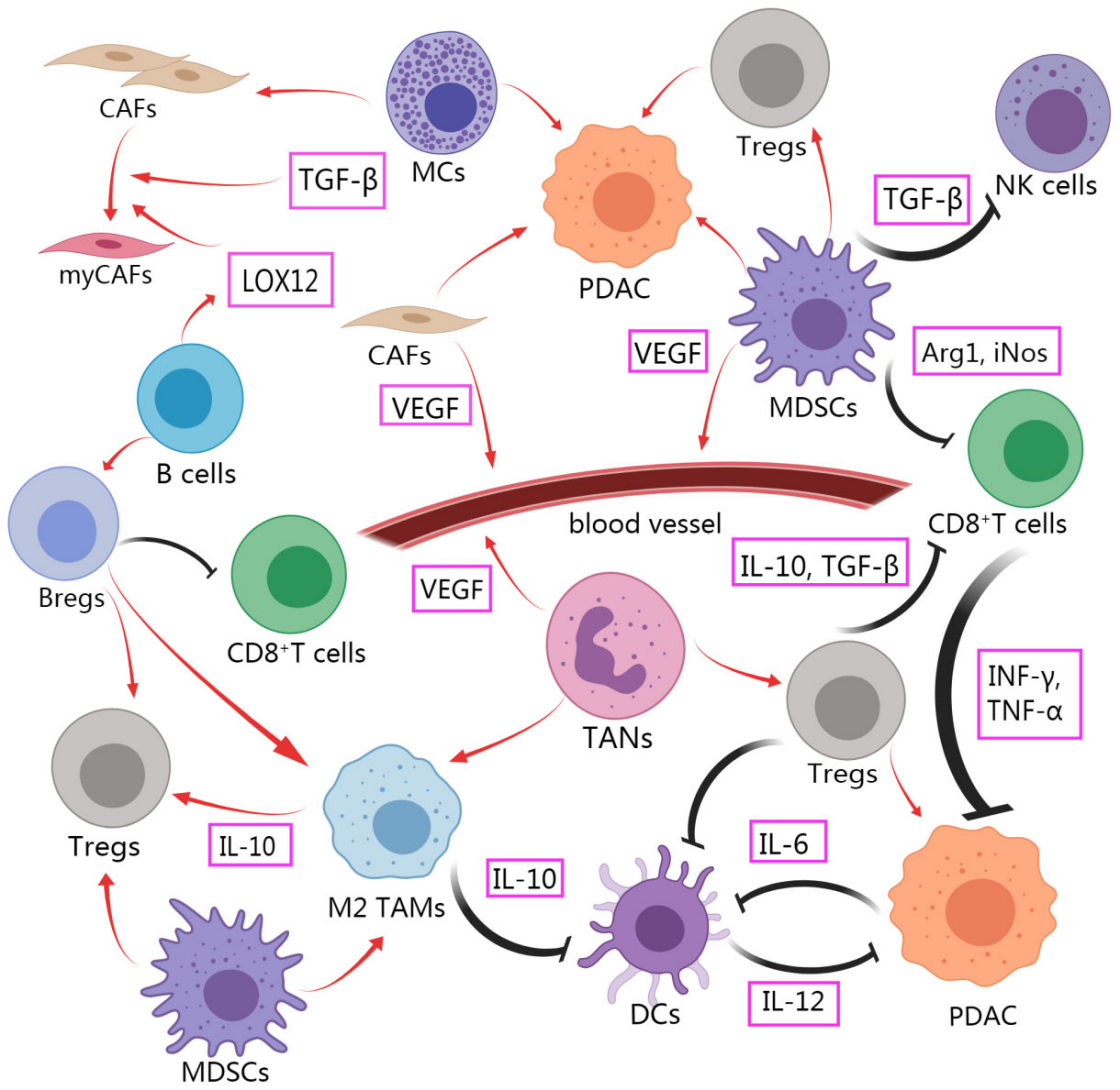
Intercellular crosstalk in TME of PDAC affects immunosuppression and tumor progression. Immunosuppressive cells promote tumor progression and play a role of functional inhibition against tumor cells. The function of anti-tumor immune cells to kill tumor is limited. Created with MedPeer (www.medpeer.cn).

The TME of PDAC contains various noncancerous cells, including cancer-associated fibroblasts, tumor-associated macrophages, tumor-associated neutrophils, myeloid suppressor cells, CD8^+^T cells, regulatory T cells (Tregs), Dendritic cells, B cells, Natural killer cells, Mast cells, Cancer stem cells (**Figure 1**). Immunosuppressive cells and other TME components weaken the therapeutic effect of innate immune cells and adaptive immune systems in PDAC. Immune support cells with invasion degree related to prognosis and survival rate are excluded or re-programmed (34). The PDAC tumor microenvironment gains immune privilege and forms troublesome immunosuppression due to the mutual interference of these cells (**Figure 2**). It is essential to comprehend the role and mechanism of these cells in the development of pancreatic cancer. By clarifying the various ways in which immunosuppression occurs in PDAC, it provides the basis for finding the methods to alleviate the immunosuppression and achieve effective immunotherapy.

### 3.1 Cancer-associated fibroblasts

Heterogeneous CAFs exist widely in the matrix of PDAC. They reshape the microenvironment by secreting extracellular matrix and produce soluble molecules and extracellular vesicles. These CAFs positively regulate immunosuppression through various mechanisms. CAFs secrete multitudinous cytokines and chemokines that promote the differentiation of macrophages into an immunosuppressive phenotype, weakening the function of immune cells and hindering the efficacy of immunosuppressive checkpoint inhibitors (35). CAFs have long been taken into account as the cells for building tumor immune escape shelters, and targeting them may be a promising therapeutic strategy. However, in recent years, many reports have pointed out that inhibiting CAFs in mouse pancreatic cancer models may lead to an increase in poorly differentiated pancreatic cancer cells and a worse prognosis. For example, genetic depletion of CAFs with high expression α-smooth muscle actin (αSMA) is faster tumor progression and a lower survival rates (36). This finding serves as an early warning that some cells in the complex CAFs family have cancer-suppressive properties. An indiscriminate attack on CAFs will promote PDAC. Single-cell sequencing was used to reveal the divergence mechanism and measure the tumor immunity of different types of branches in the CAFs group.

It is not completely sure that the origin of CAFs is PSCs, but this is the current mainstream view. Under the function of soluble molecules secreted by tumor cells, PSCs differentiate into two main subgroups with diverse distinguishing: myofibroblastic CAFs (myCAFs) with high expression of αSMA and inflammatory CAFs (iCAFs) in contrast (37). Studies have shown that myCAFs are mainly distributed around cancer cells as the main source of ECM, which has potential anti-tumor properties. On the contrary, iCAFs is far away from cancer cells and can induce inflammation in the microenvironment, which is beneficial to immunosuppression (38). Some research has identified a new type of CAFs that express MHC class II and CD74, which are called antigen-presenting CAFs (apCAFs). It has been speculated that apCAFs function in antigen presentation and bind to corresponding receptors on T cells. However, they cannot effectively stimulate T cells proliferation due to the lack of classical costimulatory molecules, resulting in immunosuppression (39). Some research have reported a kind of CAFs that have an anti-tumor effect and highly express the stem cell marker Meflin. The degree of infiltration of these CAFs is positively correlated with a better prognosis. However, the number of these cells gradually decreases as cancer progresses, leading to an increase in the immunosuppression of PDAC (40). In PDAC, the content of CAFs positive for fibroblast activation protein A (FAP) is increased, which may cause the development of cancer. FAP is a serine protease that is specific to in CAFs and assists in PDAC’s evasion of the immune system. FAP is being closely studied as a potential biomarker for diagnosis and immunotherapy target (41). Numerous cytokines stimulate the differentiation of CAFs and they can interact with each other. TGF-β relies on the Smads pathway to transmit signals that differentiate into myCAFs. Meanwhile, Interleukin-1α (IL-1α) utilizes the NF-κb pathway to increase the level of leukemia inhibitory factor (LIF), which stimulates the JAK-STAT3 signal to enhance the differentiation of iCAFs. TGF-β can inhibit the pathway that promotes iCAFs differentiation, resulting in the multiplication of myCAFs (42). CAFs differentiate into myCAFs upon receiving hedgehog signal (Hh) and into iCAFs upon receiving CXC chemokine receptor 2 signals (43, 44). Various subgroups of CAFs can convert into each other under certain conditions. P53 may play a key role in remodeling CAFs. CAFs that were originally co-cultured with P53 deficient cancer cells will transform into the dominant CAFs after being co-cultured with gain-of-function (GOF) mutant p53 (45). ICAFs and apCAFs can be transformed into myCAFs through technical cultivation. Discriminating between CAFs and other cells, as well as identifying different subtypes of CAFs, remains challenging. CAFs share common markers with certain normal cells, such as pericytes and microvascular endothelial cells. Numerous promising markers specific to CAFs have been identified as overexpressed in multiple subpopulations (46). Although there has been a breakthrough in understanding the heterogeneous of CAFs, it is difficult to elaborate on all mechanisms. Activated CAFs can recruit immunosuppressive cells. Inducing CAFs into a quiescent state may be a key strategy to improve the effectiveness of immunotherapy and reverse the immunosuppression of PDAC. Clinical trials have been conducted using drugs such as all-trans-retinoic acid (ATRA) based on this theory, in combination with chemotherapy drugs, to obtain safer evaluations (47).

Single-cell sequencing technology asserts that different CAFs play contradictory roles in PDAC. Completely inhibiting CAFs without discrimination could lead to counterproductive treatment. Ideally, we aim to support CAFs for cancer suppression and exterminate carcinogenic CAFs to obtain greater clinical benefits. Because of the complexity and mutual transformation of CAFs, the lack of biomarkers to accurately distinguish CAFs subgroups can result in to targeted drugs mistakenly injuring anti-tumor cells. It is extremely difficult to design drugs targeting CAFs. Under more arduous research, this method shows promise as a treatment for immunosuppression. It involves inducing CAFs to differentiate into tumor suppressors through reciprocal transformation mechanisms within the CAFs family.

### 3.2 Tumor-associated macrophages

Heterotypic macrophages are a prominent formation of immune cells in the microenvironment of pancreatic cancer. They have dissimilar phenotypes and functions that affect cancer progression and are considered a prognostic symbol. It is deemed that some macrophages in TME originate from bone marrow-derived classical mononuclear cells in circulation. These cells have high expression of CD14 and deletion of CD16, and they also express the immunosuppressive factor C-C Motif Chemokine Receptor 2 (CCR2) and bind to its corresponding ligand 2 (CCL2). The up-regulation of classic monocytes that can easily differentiate into tumor-promoting macrophages is associated with a worse prognosis. The macrophages of the pancreas are also derived from resident tissue macrophages generated by primitive hematopoiesis (48). In the TME, macrophages are typically classified as M1-like or M2-like based on their phenotype and biomarkers, although recent proposals suggest that this classification is too broad. M1-like macrophages express CD80, CD86, and inducible nitric oxide synthase (iNOS) on their cell surface, which has an anti-tumor effect and enhances Th1 to stimulate an immune response. After being activated by IFN-γ and TLR ligands, M1-like macrophages overexpress IL-12, IL-23, and MHC-II to control tumor growth by inducing nitric oxide synthase (49). TAMs, the major component of M2-like macrophages expressing CD160, CD206, and Arginase (ARG) -1 on the cell surface, have an immunosuppressive effect and assist Th2 in completing immune escape (50). TAMs increased gradually with the occurrence and development of malignant tumors linked to the resistance of PDAC (51). A high level of infiltration of TAMs indicates a lower overall survival rate. TAMs support tumor cell proliferation and metastasis through multiple mediators. Through a variety of cytokines and chemokines, TAMs act on tumor cells to play their own immunosuppressive role. TAMs also play an immunosuppressive role by inhibiting the function of anti-tumor macrophages. On the other hand, these cells will produce IL-10 to enhance the response of Tregs and promote Th2 to release IL-4, forming a positive feedback loop that maintains the level of TAMs (52). Inhibiting the IL-10-dependent pathway can promote the production of IL-12 by dendritic cells and alter the immune cell infiltration of PDAC to improve the prognosis (53). TAMs indirectly reduce T cell infiltration and attenuate T cell activation signal transmission through the PD-L1 site and Dectin1/galectin-9 axis (54). Additionally, Phosphatidylinositol 3-kinase γ (PI3Kγ), which is similar to the immune program switch, plays a significant role in the formation and immunosuppression of TAMs. Selectively inactivating it upregulates MHC-II and decreases IL-10 and arginase, which may become a potential method to regulate the immune response of TME to halt tumor progression (55). There is likewise a macrophage that does not express CCR2 but highly expresses endothelial receptors, which represents a worse prognosis. These cells can form new blood vessels and maintain hypoxia by combining with angiopoietin ligands (especially ANG1 and ANG2) that increase in the hypoxic microenvironment (56). According to existing cognition, macrophages are first recruited into the microenvironment and located around the vascular system. They then migrate to the hypoxic area depending on the influence of CCL2 or ANG. The infiltration of macrophages into hypoxic areas may enhance patient survival (57). TAMs in TME are frequently found surrounding the tumor vasculature, tumor cells, and other areas of malignant inflammation. On the contrary, these areas are often far away from M1-like macrophages. However, the spatial distribution of macrophages is not stable and changes as the disease progresses (49). It should also be pointed out that TAMs can be remodeled into M1-like macrophages in the presence of inflammatory factors in the microenvironment, indicating that TAMs are immune cells with remarkable plasticity potential (58). This suggests that utilizing the plasticity of TAMs to reverse the immunosuppression of PDAC could be an effective treatment. However, M1-like macrophages are not completely harmless, as they maintain a chronic inflammatory state that can promote malignant transformation (59).

### 3.3 Tumor-associated neutrophils

It generally declares that neutrophils exist in acute inflammation to perform their functions. However, in the microenvironment of PDAC, neutrophils are not only responsible for causing inflammation but also contribute to tumor proliferation and metastasis as immunosuppressive components. Tumor-associated neutrophils (TANs) are a diverse group of cells in pancreatic cancer (60). Different phenotypic characteristics represent various abilities. The heterogeneity of neutrophils plays two opposing roles in PDAC. The active molecules in TME can regulate the differentiation balance between anti-tumor subgroups and tumor-promoting subgroups. Neutrophils respond to low-dose IFN-β and transform into anti-tumor subsets that secrete CCL3, CXCL9, and other chemokines, promoting immunity by recruiting CD8^+^T cells. They also increase TNF-α and ROS expression to amplify cytotoxicity (61). Furthermore, antineoplastic neutrophils mediate the release of IL-1 and hepatocyte growth factor (HGF) to exert antibacterial activity and increase the production of iNOS to kill tumor cells, respectively (61). TGF-β induced TANs differentiation of tumor subpopulations (62). TANs interact with other cells in the microenvironment, contributing to the chronic inflammation of tumors and strengthening the immune dilemma (63). Chemokines produced by TANs, such as CCL2 and CCL17, increase the ratio of Tregs and TAMs, decrease the ratio of IL-17, and regulate the therapeutic effect of sorafenib (64). TANs generate a variety of vascular growth-promoting molecules, such as VEGF and MMP, which have a synergistic effect on angiogenesis. However, these molecules can also be targeted for inhibition as a potential therapeutic strategy. Neutrophils have been observed to increase the expression neutrophil gelatinase-associated lipocalin (NGAL) in a variety of cancers, which can inhibit angiogenesis (65).

TANs can induce tumor cell invasion and migration through various ways, in which IL-1β occupies an eminent position. In PDAC, abnormal adipocytes secrete IL-1β, which activates PSCs to recruit TANs. TANs, in turn, release IL-1β to activate PSCs, forming a positive feedback loop that promotes tumor growth (66). P2RX1 is an ion channel purinergic receptor that activates inflammation (67). In cases of PDAC with distant metastasis in the liver region, a large number of neutrophils are present with either no or low expression of P2RX1 and increased MMP-9 levels. These neutrophils activate neutrophil transcription factor NF-E2 p45-related factor 2 (NRF2) to regulate redox reaction and affect cell metabolism (68). NRF2 also induces CD8^+^T cell apoptosis through PD-L1 (69). Neutrophils regulate the content of elastase, which can destroy the E-cadherin (E-cad) of tumor cells, leading to tumor proliferation (70). CXCL5 acts as a CXCR2 ligand depends, which is dependent on NF-ĸB’s instructions during the recruitment process of TANs. Blocking this signal pathway reduces the quantity of neutrophils that induce immunosuppression (71). IL-17 increases the infiltration of TANs and inactivates therapeutic immune cells by increasing the activity of chemokines, leading to resistance to immunotherapy (72).

Neutrophil extracellular traps (NETs) can shield circulating tumor cells from immune cell cytotoxicity. These traps are secreted by neutrophils (73). NETs also mediate the epithelial-to-mesenchymal transition (EMT) (74). NETs may promote distant metastasis of tumors and could serve as a potential prognostic marker for patients (75). NETs are mainly composed of histones, which also promote angiogenesis (76).

Studying the influence and mechanism of TANs in emerging treatment methods can benefit patients. In addition to inhibiting chemokine CXCR2 to weaken the recruitment and activation of TANs, there are many new explorations (77). For instance, BL-8040, an inhibitor of CXCR4, can improve the infiltration of CD8^+^T cells and more securely alleviate the patient’s condition when combined with pembrolizumab (78). Clinical trials are being conducted on targeted inhibition of TGF-β and IL-17, which promote neutrophils to differentiate into tumor-promoting functions and collect TANs, respectively (79, 80). Targeted therapy combined with other regimens may improve tolerance and therapeutic effect. There are two main solutions to suppress NETs: inhibiting peptide arginine deiminase 4 (PADI4) to reduce its formation or exploiting DNase and chloroquine to destroy its structure (61). Thrombomodulin inhibits the transformation of epithelial cells into mesenchymal cells induced by NETs (70).

The Neutrophil/Lymphocyte Ratio (NLR) can serve as a biomarker to predict the overall survival rate of cancer, stratify the severity of patients and formulate treatment plans. This index is simple, cost-effective, and safe to operate (81). An elevated NLR level is positively correlated with a poor prognosis.

### 3.4 Myeloid suppressor cells

Pathological factors can cause immature bone marrow cells to differentiate into MDSCs along with bone marrow progenitor cells. MDSCs, like other immune cells, are heterogeneous and cause immunosuppression by regulating various mechanisms (82). These cells are mainly divided into monocyte subsets (M-MDSC) and polymorphonuclear cell subsets (PMN-MDSC) as they are composed of immature macrophages and immature granulocytes. The proportion of the latter is higher than that of the former, but the immunosuppressive function of the former is more important (83). Tumor cells regulate the aggregation of MDSCs to tumor sites by releasing chemokines CCL2 and CCL5 (84). Growth factors such as GM-CSF and G-CSF also participate in the recruitment of MDSCs (85). TNF-α、IL-6 and IL-1β contribute to the recruitment of MDSCs by acting on other immune cells. Prostaglandins PGE2 and IL-17 can increase the number of MDSCs and promote them to produce more ARG1 to maintain immunosuppression (86). Sphingosine-1-phosphate (S1P) is expressed more in the hypoxic microenvironment of PDAC and recruits MDSCs (14). The content of MDSCs in pancreatic cancer changes with disease progression. MDSCs are activated by VEGF from stromal cells and can also secrete VEGF to further enhance their activity (87). MDSCs play an extremely prominent role in the immunosuppression of PDAC and are related to a variety of tumor-promoting cells and bioactive molecules. Because of the vascular collapse of the matrix and the high-pressure environment, the nutrients available for immune cells supplied by TME are quite scarce. MDSCs inhibit the proliferation and function of surface receptors of CD8^+^ T cells by producing ARG1 and iNOS, which consume nutrients by utilizing L-arginine (88). Arg1, iNOS, and NADPH oxidase 2 (NOX2) have oxidative activity that inhibits the tumor immune response by enhancing the content of NO and ROS (86). MDSCs can adjust the content of indoleamine 2,3-dioxygenase (IDO), which degrades tryptophan. With the increase of IDO content, there are more toxic metabolites in the microenvironment. MDSCs also resist tumor immunity by enhancing the expression of PD-L1 (89). Similarly, MDSCs release TGF-β and IL-10, which block the effect of natural killer cells. The mechanisms of immunosuppressive effects of MDSCs can induce T cells to differentiate into Tregs, indirectly assisting tumor immune escape by increasing Tregs invasion (86). MDSCs are also associated with the decrease of anti-tumor T cell content in precancerous lesions is also associated with the role of MDSCs. MDSCs can also improve the differentiation ratio of TAMs (14). Cooperating with other immune cells, MDSCs produce VEGF and MMP9, which are conducive to tumor angiogenesis (90). MDSCs promote tumor cells to invade and migrate outward by affecting the pre-metastasis niche.

MDSCs can cause immunosuppression through various mechanisms, but these effects may not be universal among patients. Despite this, the significant infiltration of MDSCs in PDAC patients leads to resistance to various treatment strategies and a worse prognosis. In recent years, many therapeutic approaches targeting MDSCs have been put into animal models. One potential strategy is to encourage the differentiation and maturation of MDSCs. Trans-retinoic acid is proposed for use due to its ability to induce differentiation. Animal models and patient studies have shown that this drug can effectively reduce MDSCs (91). The immunosuppressive pathway targeting MDSCs is also available. Inhibition of PGE2 can restore the tumor immune response (92). These therapeutic methods that inhibit MDSCs are conducive to enhancing the therapeutic effect of immunotherapy targeting PD-1 (93). Lactic acid can regulate the tumor-promoting function of MDSCs and alleviate the immunosuppression of TME under radiation stimulation (94). The combined treatment of GM-CSF, which blocks the recruitment of MDSCs and gemcitabine reduces the differentiation ratio of MDSCs decreases and improves the function of anti-tumor immune cells (95). We speculate that inhibiting MDSCs will effectively alleviate the immunosuppressive characteristics of PDAC and promote the effect of immunotherapy.

### 3.5 T cells

The TME contains complex T cells, including CD8^+^T cells and CD4^+^T cells. CD8^+^T cells play a cytotoxic role and are also known as cytotoxic T cells, while CD4^+^T cells can be further classified as Th1, Th2, Th17, and Tregs (96). Gene mutations in TP53, CDKN2A, and SMAD4 occur during the progression of pancreatic precancerous lesions to cancer. Mutated genes that cause chronic inflammation of TME aggravate tissue loss by activating CAFs, increasing VEGF release, and mediating the NF-ĸB pathway. These genes are also associated with the inhibition of cytotoxic lymphocytes (CTL) and an increase in Tregs (97).

CD8^+^T cells are predominantly distributed in the microenvironment far away from tumors. Moreover, under the effects of various immunosuppressive cells, the number of activated cells and the infiltration of CD8^+^T cells are reduced. PDAC is typically classified as a cold tumor that is difficult to elicit an immune response (98). However, studies have shown that surgical treatment can increase infiltration of CD8^+^T cells in PDAC patients, indicating their potential as an anti-tumor agent (99). The distribution and degree of T cell infiltration have been linked to different phases of pancreatic cancer. The general trend is that CD8^+^T cells decrease gradually and are mainly distributed in the tumor margin and dense matrix, while Tregs increase gradually and are mainly distributed around the tumor cells (100). The dense matrix restricts CD8^+^T cells from approaching and clearing the tumor. CXCL12 can mediate the migration of CD8^+^T cells out of the marginal region (101). The prognosis of patients improves with an increase in CD8^+^T cells in close proximity to tumor cells. CD8^+^T cells eliminate tumor cells through the release of granzyme and perforin. The infiltration of granzyme B^+^ CD8^+^ T cells represents longer survival time. CD8^+^T cells release anti-tumor mediators such as TNF-α, and IFN-γ to destroy abnormal cells (14). The formation of massive lymphocyte aggregation to form tertiary lymphoid structure (TLS) represents a better prognosis (102). The low mutation rate of PDAC results in a lack of CD8^+^T cells and few new antigens to induce an effective immune response (103). The immunosuppressive programmed death receptor (PD-1) and corresponding ligand (PD-L1) are up-regulated on the surface of CD8^+^T cells, leading to their weakening primarily through the Fas-mediated apoptosis pathway (104). The quality of T cells is more important than their quantity. Under these mechanisms, the reduced number of CD8^+^T cells is difficult to activate and presents a depletion phenotype.

CD8^+^T cells are activated by Th1 cells through the secretion of IFN-γ to facilitate tumor immunity (105). The immune infiltration of Th2 cells can induce the secretion of cytokines that inhibit immunity and promote the differentiation of TAMs (106). Th17 cells produce IL-17 and also play an immunosuppressive role (98). During the early stages of tumor occurrence, Tregs infiltrate both the tumor and circulation (101). Tregs overexpression of CTL-associated antigen 4 (CTLA-4), which inhibits antigen-presenting cells from expressing costimulatory ligands CD80 and CD86 for activated T cells (107). Similarly, some CAFs bind to CD8^+^T cells without activation. Tregs also mediate a variety of signal pathways, such as IL-10 and TGF-β, to prevent the immune function of CD8^+^T cells (14).

Continuous exposure to tumor antigens can cause T cells to become exhausted, reducing their proliferation ability and increasing the expression of inhibitory receptors such as PD-1 and CTLA-4 on their surface, transforming into exhausted T cells. These exhausted T cells can be divided into two subgroups: the incompetence subgroup and the aging subgroup (108). Cytokines like TGF-β and IL-2 can stimulate the increase of PD-1. PD-L1 and PD-L2 ligands of PD-1 come from a variety of cells, including tumor cells, TAMs, and MDSCs. Their contents are positively regulated by IFN-γ. The PD-1 pathway impedes the dephosphorylation of the T cell surface receptor CD28, leading to its inactivation (109). CTLA-4 interferes with costimulatory receptors, thereby restraining CD28 (110).

At present, extensive research has been carried out to restore and utilize the immune function of CD8^+^T cells. But PDAC does not benefit as much as other cancers. Various research directions exist based on the mechanism of T cells in tumors, including ICB, chimeric antigen receptor (CAR) T cells, and tumor vaccines. At present, the targets of immunotherapy for pancreatic cancer are PD-1 and CTLA-4. However, due to the presence of rare mutations and new antigens in PDAC, as well as the complex relationship between internal immunosuppression is complex, these treatments have not shown significant therapeutic gains (111). Gemcitabine is the first choice chemotherapy drug for PDAC. It has been shown to inhibit the immunosuppressive effect of Tregs (112). Combining multiple therapies that target the mechanism of T cell immunosuppression may be a promising direction for future research. The mechanism by which T cells induce immune escape and weaken the anti-cancer effect requires further consideration and exploration.

### 3.6 Dendritic cells

Dendritic cells (DCs)play a crucial role in innate and adaptive immunity by recognizing antigens, transmitting signals to T cells, and activating the immune response of T cells. As antigen-presenting cells (APCs), DCs exist in tumors as the first step to capture and process tumor cells (113). DCs are a heterogeneous cell population that can be classified into conventional DCs (cDCs) and plasma cell-like DCs (pDCs) according to different classification methods. The former can be subdivided into two types of dendritic cells: BDCA3^+^cDC1s, which activate CD8^+^T cells, and BDCA1^+^cDC2s, which transmit signals to CD4^+^T cells. Ideally, BDCA3^+^cDC1s can induce a T cell immune response in the microenvironment under the influence of CCL2 (114). But in PDAC, immunosuppressive components exist widely in the microenvironment. The hypoxic and nutrient-deficient microenvironment makes it difficult for DCs to play a strong immune function. Immunosuppressive cells and their products hinder the proliferation and complete activation of DCs. VEGF and IL-6 from tumor cells impair the function of DCs (115). TAMs and Tregs overexpress IL-10, which down-regulates the content of IL-12 released by DCs, thereby limiting the stimulation of the tumor immune response (107). TGF-β and PD-1 strengthen the inhibition of effector T cells by limiting the function of DCs. Immunosuppression-controlled DCs promote a reduction in Th1 differentiation and an increase in Th2 differentiation among CD4^+^T cells (116). This hinders the smooth execution of the initial stage of the tumor immune response and affects the entire anti-tumor mechanism network. Therefore, DCs have more functions in promoting immunosuppression and drug resistance. DCs can also be separated into CD11-positive myeloid DCs and CD11-negative lymphoid DCs. The medullary DCs’ high degree of infiltration results in a longer survival time (117). Treatment strategies based on improving DCs are also being tried.

### 3.7 B cells

PDAC is a heterogeneous cell population that contains numerous B-lymphocytes. The relationship between B cells and tumors is very complex, containing both promotion and inhibition of tumors. B cells can destroy tumors by producing antitumor antibodies and presenting antigens to T cells. However, some subsets of B cells also promote tumor growth by performing immunosuppressive functions (118). B cells have been observed in pancreatic precancerous lesions. Stromal cells regulate B cells through the chemokine CXCL13, which leads to their accumulation in tumors as cancer progresses (119). The hypoxia-inducible factor HIF1a forms and maintains the anoxic state of TME. Inhibiting HIF1a activity can increase CXCL13 to recruit more B cells (120). Alleviating B cells exhaustion can mitigate the development of PDAC, further highlighting the significant role of B cells in the occurrence of PDAC. The spatial distribution of B cells may influence the prognosis. B cells distributed in the matrix have been associated with terrible tumor outcomes, potentially due to increased expression of PD-L1 and TGF-β. Conversely, B cells distributed in ectopic lymph node-like TLS have been related to the prolongation of survival times, possibly due to improved capability of B cell proliferation and activation (121). The poor immunogenicity of PDAC not only makes it difficult to activate cellular immunity but also affects humoral immunity. Anti-cancer autoantibodies secreted by B cells are only found in a minority of cases (approximately 30%) (122). This makes it difficult for the humoral immune response to achieve therapeutic objectives. B cells can promote the matrix formation of TME. B cells promote the fibrogenic properties of CAFs by secreting cytokines such as PDGF-β (123). Under the influence of B cells, CAFs increased collagen expression and enhanced their ability to remodel epithelial cells into stroma. Circulating plasmablasts, which are the best at expressing of this ability in heterogeneous B cell populations, not only activate CAFs but also secrete LOXL2 that acts on collagen (124). LOXL2 enhances the stretching ability of collagen and regulates the differentiation of fibroblasts into myCAFs (125). This type of cell population is rarely observed in healthy people. On the contrary, circulating plasmablasts are specifically present in the peripheral blood of PDAC patients, especially in untreated patients. These plasmablasts are continuously exposed to tumor antigens in circulation (126). Based on this unique existence, circulating plasmablasts may become a marker for the diagnosis and prognosis of PDAC. It is unclear whether B cells consistently promote or inhibit the formation of the fibrotic matrix. In B cells deficient PDAC mouse model, the fibrogenic property is significantly weakened, showing relatively slowing tumor progression (122).

Regulatory B-cell subsets (Bregs) in B cells mainly play a role in tumor promotion. THE increased infiltration of Bregs in TME leads to poor prognosis and low survival rates (127). Bregs cause immunosuppression and drug resistance by promoting Tregs production, inhibiting the production of Th1 and inflammatory factors, and inducing the anti-inflammatory transformation of antibodies (122). Bregs interact with PDAC cells, and tumor cells release IL-18 to promote the production of Bregs (128). Bregs overexpress PD-L1, bind to CD8^+^T cells in an inhibitory manner, and reduce the secretion of IFN-γ by CD8^+^T cells, thereby assisting PDAC in escaping immune surveillance (129). IL-10 and IL-35 released by Bregs decrease the level of IFN-γ and increase the number of Tregs and tumor cells (130). Bregs secrete Bruton tyrosine kinase (BTK), inducing the differentiation of immunosuppressive TAMs in TME (131). The immunosuppressive properties of B cells in PDAC stimulate the exploration of new therapeutic methods. Targeted therapy based on the cancer-promoting mechanism of B cells may be crucial in improving the survival rate for pancreatic cancer. Evidence suggests that heterogeneous B cells contain tumor-inhibiting subsets (132). Non-selective exclusion of all types of B cells is not feasible. Precisely localizing and selecting patients with indications has become a huge challenge for therapeutic methods targeting B cells. If the B cells that constitute the immunosuppressive properties of PDAC are precisely blocked, this will provide a better working environment for immunotherapy.

### 3.8 Other ingredients

As a momentous member of the innate immune system, NK cells take advantage of cytotoxicity to fight against tumors in the early stages of tumor development. However, NK cells exhibit varying degrees of infiltration in the microenvironment depending on the type of tumor. In PDAC, low-invasive NK cells are confined to the tumor periphery and have limited contact with tumor cells. Nevertheless, it has not been determined whether there is a significant relationship between the infiltration degree of NK cells and the prognosis of patients (133). NK cells require mediation by cell surface receptors to exert their anti-tumor activity. If the TME of PDAC contains numerous ligands that activate NK cells, such as CCL2, it can attract NK cells to infiltrate the tumor. The infiltration of NK cells can affect the prognosis of tumors through this mechanism. NK cells have the ability to eliminate tumor cells and identify and kill tumor stem cells. The anti-tumor function of NK cells is magnified when chemotherapy drugs are applied, resulting in a decrease in the recurrence rate of PDAC mice (134). In PDAC, a suppressed IFN-γ pathway can affect the recruitment of NK cells and lead to a poor prognosis (135). Like CD8^+^T cells, NK cells also nutrient-deficient and suppressed in their anti-tumor function. Therefore, exploring immunotherapy to enhance the function of NK cells is a focus.

Usually, people take into account the allergic properties of Mast cells (MCs) to cause diseases. However, recent studies have shown that MCs also exist in the occurrence and progression of tumors, providing support at different stages. These cells are often widely distributed in the skin, respiratory tract, and visceral mucosa. MCs migrate to TME under the action of chemokines such as VEGF in TME. MCs present in tumors have been given a new concept: tumor-associated mast cells (TAMCs) (136). The distribution of MCs in tumor tissue is inconsistent, with a notable absence at the center of the tumor tissue. The degree of MCs infiltration is negatively correlated with a good prognosis (137). MCs secrete soluble factors such as TNF-α, TGF-β, MMP, IL-8, IL-13, VEGF, histamine, and tryptase, which affect the proliferation, activation, and function of other cells in the microenvironment (138). Under the guidance of the above cytokines, MCs accelerate the expansion, diffusion and migration of tumor cells, promote the formation of blood vessels and lymphatic vessels (139). MCs can promote the proliferation of PSCs and CAFs to form the dense matrix of pancreatic cancer. MCs play an effective role in recruiting MDSCs and can block the therapeutic effect of chemotherapy drugs (140). Controlling the tumor-promoting mechanism of MCs can retard tumor progression. Some studies have indicated that MCs play an anti-tumor role by secreting IL-33 (141).

Cancer stem cells (CSCs) are a group of cells that can self-renew and differentiate like stem cells. They mix with tumor cells and exist in the microenvironment. CSCs of PDAC increase the carcinogenic effect, which is characterized by reaching the markers such as CD44 and CD24. Patients with high CD44^+^CSCs content have a worse prognosis. These cell surface markers can be stably expressed in the offspring (55). This mechanism maintains the immunosuppressive effect of CSCs. The proliferation, differentiation, and resistance therapy of CSCs benefit from anoxic acidic TME. Tumor cells located in an appropriate niche under hypoxia are prone to maintain their stemness, including an undifferentiated state, self-replication, and migration (142). CSCs with different phenotypes are subject to different degrees of hypoxia and chemical action (143). CSCs not only promote cancer cell proliferation but also metastasis through nestin, a protein marker. TGF-β increases the content of nestin under anoxic conditions and plays a positive role in regulating TGF-β (144). At present, treatment methods for CSCs are not yet mature.

Microbiota is often ignored in pancreatic cancer, but they can promote the occurrence and development of pancreatic cancer. By comparing normal pancreatic tissue, researchers found that bacterial DNA and 16S rRNA increased in PDAC tissue. Microbiota affects the TME of PDAC. The number of CD8^+^T cells and M1-like macrophages in TME of PDAC increased and the number of MDSCs decreased after inhibition of the microbiota by antibiotics. The expression of bacterial lipopolysaccharide was positively correlated with PD-L1. Oral antibiotics and anti-PD-L1 immunotherapy have synergistic effect. These effects ultimately lead to the slowing down of PDAC progression in the mouse model. However, the benefits of this combination therapy for patients with PDAC are controversial. Other tumor patients showed poor prognosis caused by antibiotics before immunotherapy. These effects ultimately lead to the slowing down of PDAC progression in the mouse model. However, the benefits of this combination therapy for patients with PDAC are controversial. Other tumor patients showed poor prognosis caused by antibiotics before immunotherapy. Other immunotherapies, such as cancer vaccines and adoptive cell therapy, may also involve microbiome responses. In PDAC, the relationship between microbiota and immunosuppression needs further exploration (145).

## 4 Immunosuppressive molecules and targeted therapy

In the PDAC microenvironment, various cytokines and signal pathways regulate the tumor immune response in addition to the immunosuppressive cells and molecules mentioned above. The TME features a close interaction between various cells and soluble molecules to ensure the immunosuppression of PDAC. Further exploration of these soluble factors may identify them potential targets for anti-tumor therapy. Some of these factors will be explained in detail below. **Table 1** summarizes other soluble molecules and signal pathways related to immunosuppression and their corresponding therapeutic drugs. **Table 2** summarizes all the cells and soluble factors mentioned in the article that have dual effects of immunosuppression and anti-tumor in TME.

**Table 1:** Some cytokines and targeted drugs related to immunosuppression.

Core substance	Action mechanism	Effects on immune- cells	Effects on immunosuppression	Refs.
GOT2	GOT2-PPARδ Axes: combining and activating PPARδ Nuclear receptor	Restraining function of CD8^+^T cell	Promoting	(146)
N6L: Pseudopeptide inhibiting NCL (nucleolar protein)	Inhibiting tumor angiogenesis, reducing the level of IL-6, and reducing the formation of matrix by CAFs	Reduce Tregs and MDSCs, and increase TILs	Inhibiting (N6L) Promoting (NCL)	(147)
VDR	Hindering matrix production by PSCs	Promoting the contact of anti-tumor cells with PDAC cells	Inhibiting	(148)
miRNA-155	Suppressing the expression of SHIP-1	Adding TAMs	Promoting	(149)
Hsa_circ_0046523	Increasing the content of IL-10 and TGF-β, reduce the content of IFN-γ and IL-2, and promote the expression of PD-L1	Increasing Tregs and suppressing the infiltration and function of CD8^+^T cells	Promoting	(150)
LL-37 (the human cathelicidin peptide)	Inhibiting autophagy of PDAC cells, increasing ROS production, inducing DNA damage and cell cycle arrest	Reducing MDSCs and TAMs, and increasing CSD8^+^T cells	Inhibiting	(151)
UQCRC1 (Components of mitochondrial complex III)	Reducing the expression of CCL5 and changing the number of DNAM-1 and CD96 receptors on NK cell surface through UQCRC1/eATP axis	Suppressing the infiltration and function of NK cells	Promoting	(152)
IRAK4	Promoting matrix fibrosis through NF-κB pathway	Leading to depletion of anti-tumor T cells	Promoting	(153)
MNKs inhibitor	The increase of MNK activity is related to the decrease of CD8^+^T cell infiltration. Repression of this pathway increases the expression of immunosuppressive markers in TAMs	Increase CD8^+^T cells, but induce T cell depletion and enhance the ability of TAMs	Promoting	(154)
BMS-687681 (CCR2/CCR5 inhibitor)	Suppressing the tumorigenic effect of CCR2/CCR5 and blocking the signal pathway of TLR2/4 and RAGE	Inhibiting Tregs, TAMs and MDSCs as well as increasing anti-tumor T cells infiltration	Inhibiting	(155)
LIPH (lipase H)	Its high expression is related to mutations of KRAS, TP53, CDKN2A and SMAD4, and promotes EMT and angiogenesis	Increasing TAMs and Tregs, decreasing CD8^+^T cells and Th1 cells	Promoting	(156)
DCLK1	Advancing EMT	Increasing TAMs, reducing CD8^+^T cells, and losing E-cad	Promoting	(157)

**Table 2:** The cells and molecules in the table not only help PDAC to form immunosuppressive properties, but also have potential anti-tumor immunity.

Cell or molecule	Immunosuppressive	Antitumor effect	Refs.
myCAFs	Generating ECM to form dense matrix in TME	Potential anti-tumor properties	(37, 38)
M1-like macrophages	Maintaining chronic inflammatory state and promote malignant transformation	Expressing CD80, CD86 and iNOS, and enhancing the function of Th1	(49, 59)
TANs	Inducing chronic inflammation of tumor and increasing the proportion of Tregs cells and TAMs	Recruit CD8^+^T cells and up-regulating the expression of TNF-α and ROS	(61, 63)
Tregs	Suppressing costimulatory ligand, mediating IL-10 and TGF-β to inhibit CD8^+^T cells	The degree of infiltration of the subgroup expressing FOXP3 was positively correlated with the survival rate of patients	(158)
MCs	Promoting tumor and matrix, inhibiting anti-tumor cells	secreting IL-33	(137, 141)
Notch	Promoting vascular remodeling	Promoting aging of CAFs, differentiation of macrophages with M1 phenotype and activation of CD8^+^T cells	(55, 159)
TGF-β	cooperating with PD-1/PD-L1 to inhibit anti-tumor cells	Promoting Smad3 to Smad4 connection to transmit anti-tumor signals	(160, 161)
STING	Mediating IL-35 to promote the proliferation of Bregs and indirectly promoting the control of Bregs on NK cells	Inducing innate immune system and expressing IFN	(162)
TNFR2	Mediating NF-κB pathway to increase the level of PDL1, inhibiting cancer immunogenicity and accelerating tumor growth	Its increasing number related to the prognosis with high infiltration of CD8^+^T cells	(163)
adipocyte	Activating PSCs and recruiting TANs	Activating anti-tumor immunity by high adipocyte infiltration	(66, 164)
EVs	Serving as carrier to complete intercellular crosstalk in microenvironment	Potential therapeutic targets and safer drug carriers	(165, 166)

Notch signaling mainly exists in PDAC as an immunosuppressive component. It has multiple functions (1): regulating tumor angiogenesis. This pathway indirectly promotes the vascular remodeling of PDAC by increasing the number of endothelial cells secreting Jag1 (159). Inhibition of this pathway negatively regulates angiogenesis and tumor progression. (2) contributing to tumor cells getting rid of dormancy. (3) regulating the development, differentiation, and function of lymphocytes, and (4) accelerating the aging rate by promoting CAFs, differentiating M1-like macrophages, and activating CD8^+^T cells to promote tumor immunity (55).

TGF-β promotes tumor progression and metastasis, and may also induce tumor cell apoptosis. It can combine with the PD-1/PD-L1 inhibitory signal pathway to reshape the microenvironment and affect the proportion of immune cells in TME. This results in a reduction of CD8^+^T cells and CD4^+^T cells with Th1 phenotype, and an increase in Tregs. The combination of TGF-β and ICB inhibitors improves the infiltration of anti-tumor T cells and the level of IFN-γ (160). TGF-β exercises tumor resistance by promoting the transcription factors Smad2 and Smad3 to connect Smad4 to transmit signals (161). Theoretically, it is not advisable to completely obstruct the TGF-β pathway, as deleting this pathway or the Smad gene may result in tumor promotion.

Focal adhesion kinase, which is highly expressed in PDAC, is phosphorylated to regulate matrix deposition and assist in immunosuppression. FAK reduces the number and activity of anti-tumor cells by promoting the production of type ǀ collagen and mediating CCL5 to increase the infiltration of Tregs (167). FAK is a promising anti-tumor target and prognostic marker. Inactivation of FAK has shown to decrease the degree of PDAC fibrosis and the number of immunosuppressive cells (168). However, following the application of the FAK inhibitor, PDAC mediates matrix depletion and STAT3 signaling to down-regulate TGF-β/Smad pathway functions to form drug resistance (169).

The Hh signal primarily originates from CSCs, which mediate NF-ĸB and promote the production of tumor growth factor at the high expression levels. Inhibiting this pathway can suppress the stem cell-like characteristics and immunosuppression of CSCs (170).

Over-activation of Wnt/β-catenin causes the activation of oncogenes which exist in all stages of PDAC and play an immunosuppressive role (171). Specifically, β-catenin has been found to inhibit T cell infiltration, activation, and function, while promoting tumor invasion, metastasis, and drug resistance of tumors (172). CD4^+^T cells influence other kinds of T cells in PDAC by mediating the elevated expression of TCF7, which is also involved in the Wnt signaling pathway. Selective elimination of TCF7 increased CD8^+^T cells and decreased Tregs. Intercepting the Wnt/β-catenin signaling pathway can alleviate the immunosuppression of pancreatic cancer. However, it is important to consider the potential toxicity of WNT inhibitors and the limited treatment time window (173).

The Hippo signal pathway can promote the recruitment of TAMs and MDSCs and increase the effect of immunosuppressive cells (174).

High levels of the proliferative marker Ki67 leads to subtle infiltration of anti-tumor T cells. This may certify that cell cycle inhibitors can regulate the proliferation of ductal cells and combined with immunotherapy to create a new therapeutic approach for PDAC (86).

SUMOylation is a reversible modification observed in PDAC during the cell cycle. An inhibitor that blocks this process from pharmacology is TAK-981, which has precise selectivity and effectiveness. Upon application of TAK-981, the G2/M cell cycle arrests and chromosome segregation fails, leading to a safe reduction in tumor load by causing tumor cells from completing mitosis. In addition, TAK-981 is also conducive to the activation of CD8^+^T cells and NK cells (175).

MARCO is a scavenger receptor found on the surface of macrophages. It affects anti-tumor lymphocyte infiltration and predicts a poor prognosis. This substance is highly expressed by TAMs under the command of IL-10 and the hypoxic microenvironment. MARCO reduces the infiltration of CD8^+^T cells and NK cells in PDAC and inhibits effective anti-tumor immunity. The utilization of MARCO-targeted antibodies induces the repolarization of TAMs, improves the level of IL-18, and strengthens the function of anti-tumor cells (176).

Sphingomyelin synthase 2 can play a positive role in regulating CSF1R/STAT3 signal pathway. Inhibiting the enzyme with YE2 reduces CSF1R levels and controls macrophage differentiation into the M2 phenotype. Deletions of sphingomyelin synthase 2 or utilization of YE2 can inhibit tumor progression by weakening the invasion and function of TAMs, which may be a promising-targeted treatment scheme (177).

The STING (Stimulator of Interference Genes) signal induces the innate immune system to resist tumor invasion and express IFN through the cGAS-STING Signaling Pathway. However, the application of STING agonists mediates IL-35 to promote the proliferation of Bregs and indirectly suppresses NK cells activity. This result leads to fewer NK cells and weaker anti-tumor function, which is counterproductive. STING agonists are often used in combination with other therapies to ameliorate their efficacy on PDAC (162).

The production of Siglec-15 by macrophages promotes the differentiation of macrophages into the M2 phenotype. Siglec-15 has a negative impact on cGAS-STING Signaling Pathway. The secretion of Siglec-15 is regulated by IL-4. When Siglec-15 is suppressed, the expression of TAMs surface markers such as CD206 and Arg1 decreases. Targeting Siglec-15 may be a novel way to curb the growth of PDAC (178).

Histone modification is an important process of tumor proliferation. High levels of histone deacetylases (HDAC) can inhibit the transcription of tumor suppressor genes, leading to tumor development. More than 50% of PDAC expressed HDAC 1, which was associated with lower overall survival. HDAC 3 promotes the expression of PD-L1. HDAC inhibitors act on both PDAC cells and TME. The overexpression of HDAC in PDAC increases the recognition probability of HDAC inhibitors on cancer cells. HDAC inhibitors destroy the growth and differentiation of PDAC cells, and ultimately lead to cancer cell death, while non tumor cells are rarely affected. HDAC inhibitors also regulate VEGFA and HIF1A to prevent angiogenesis. However, the anti-vascular effect may lead to the difficulty of drug transportation to the tumor. HDAC inhibitors are expected to change the immunosuppressive properties of PDAC. HDAC inhibitors increase the antigen-presenting function and the expression of costimulatory molecules, which are beneficial to the activation and maintenance of T cells. In the preclinical model, the combination of HDAC inhibitor and anti-PD-L1 therapy successfully enhanced the anti-tumor efficacy (179).

## 5 Immunotherapy of PDAC

Patients with malignant tumor PDAC often miss the optimal time for surgical treatment because of the difficulty of early diagnosis. PDAC has high drug resistance and poor sensitivity to chemotherapy and radiotherapy. Immunotherapy is a potential strategy to ameliorate the prognosis of PDAC patients (180). The TME of PDAC has the rich and dense matrix and complex immunosuppressive cells and factors, which make an immune response difficult to occur and complete the immune escape of PDAC. New exploration focuses on destroying the mechanism of immunosuppression in PDAC and increasing the activation and function of immune effector cells. Combining immunotherapy with other conventional therapies may also yield better results for patients.

### 5.1 Immune checkpoint blockade

PDAC has two fundamental signal pathways, PD-1/PD-L1 and CTLA-4/B7,that prevent T cells from performing their immune functions. These pathways serve as the immune checkpoint of classical immunotherapy. PD-1 is an immune cell surface receptor that is up-regulated in PDAC. It combines with PD-L1 from PDAC cells to obstruct the generation of an effective immune response (181). In order to restore the proliferation, migration, and anti-tumor activity of CD8^+^T cells, it is advantageous to repress the PD-1/PD-L1 signal pathway using drugs such as nivolumab, durvalumab, atezolizumab, avelumab, and pembrolizumab. The combined inhibition of PD-1/PD-L1 and chemokines CXCR4 or CXCL12 is also safe and achievable, which can increase the infiltration of T cells and the occurrence of cytotoxic reactions (182). The level of CCL5 is indirectly affected by the overexpression of PD-L1, and co-inhibition of both can reduce the recruitment of Tregs and increase the anti-tumor effect (183). There is further evidence that the combined inhibition of PD-L1 and BTK has not yet brought more significant benefits (184). Blocking the CTLA-4 pathway with ipilimumab and tremelimumab can reduce competition with CD28, allowing for more immunostimulatory molecules to combine on the surface of immune cells. This weakens the immunosuppression of Tregs and enables expansion and utilization of more anti-tumor-T cells (185). The TME of PDAC contains a variety of vasopromoting substances. The combination of anti-angiogenesis therapy, such as Bevacizumab and Axitinib, with ICB not only ameliorate the immune response but also perfect the microvascular structure. Factors that may influence the therapeutic effect of ICI include high lymphocyte infiltration in the tumor, increased of PD-L1 levels, and high tumor mutation rates (31, 186). In addition to positive effects, toxicity and adverse events of immunotherapy should also be considered. This will promote us to study safer treatment strategies.

### 5.2 Cancer vaccines

DCs, as APCs, are unable to complete their necessary functions in PDAC, which hinders the activity of anti-tumor T cells. At present, research on tumor vaccines mainly focuses on DCs (187). DCs are obtained from PDAC patients through leukocyte isolation technology. Tumor antigens are sometimes added to strengthen the function of activating T cells before the DCs vaccine is returned to the patients. The DCs vaccine is designed to be used together with anti-angiogenic drugs, which may be instrumental in the long-term survival of patients (188). In addition, it includes whole-cell and DNA/peptide vaccines. GVAX, one of the tumor vaccine therapies for whole tumor cells, promotes the recognition of APCs and absorbs tumor antigens by expressing GM-CSF (189). The KRAS vaccine is a type of peptide vaccine. Although the specific vaccine treatment in clinical trials is still being adjusted, it is feasible to ameliorate the patient’s condition of PDAC (23). MRNA vaccine is a new and promising vector for the development of personalized vaccines against PDAC. The mRNA vaccine overcomes the heterogeneity of the tumor and meets the requirements of precise targeting, efficiency, safety, and economy in treatment(Personalized pancreatic cancer therapy: from the perspective of mRNA vaccine). Recently, the research on the combined application of tumor vaccines and ICB is under excavation.

### 5.3 Adoptive cell therapy

CAR T cells, as a new treatment for pancreatic cancer, have attracted much attention. By modifying patients’ T cells, the newly generated CAR T cells carry receptors that specifically recognize and bind tumor cell antigens and can transmit signals to induce T cells activation. The latest CAR T cells also increase the expression of costimulatory receptors (190). However, it is complex and arduous for CAR T cells to precisely target specific antigens of tumor cells. Adoptive cell therapy targeting the cell surface marker CD19 has been demonstrated to carry a high risk of tumor recurrence and acute B cells leukemia or B cells lymphoma (191). In order to effectively apply this expensive treatment strategy, the durability of CAR T cells is required. They potentially interfere with normal cells. Therefore, CAR T cells with suicide genes were proposed and examined (192). The targets entering the clinical trial stage include mesothelin(MSLN), CD133, prostate stem cell antigen (PSCA), Claudin 18.2, HER2, MUC1, carcinoembryonic antigen (CEA), etc (193). The great challenge of adoptive cell therapy is not only to prevent CAR T cells from reacting with non-target cells but also to promote their penetration of the dense matrix environment and contact with cancer cells. CAR NK cells can be used as anticancer drugs in solid tumors. NK cells are easy to obtain. Them will not cause serious side effects in allogeneic injection. At present, most trials on CAR NK cells are in preclinical or early clinical trials. The development of CAR NK cells requires more complex and more optimized transfection techniques (194). TCR T cells need to extract individual peripheral blood mononuclear cell samples and screen out the samples that can selectively bind to TCR sequence of target antigen. After the T cells in the patient’s peripheral blood are then genetically engineered and injected to eliminate tumor cells. The fourth generation of TCR T cells is a highly specific cellular immunotherapy for new antigens, with a higher safety profile. Some cases have proved that PDAC patients experience objective regression after using TCR T cells (195). Compared to CAR T cells, TCR T cells have better prospects because they are easier to enter the interior of solid cancer. Despite poor persistence, significant adverse reactions, and high consumption, adoptive cell therapy is increasingly suitable for clinical trials as it undergoes continuous improvement. Combining adoptive cell therapy with other therapies may be a promising anti-tumor method.

### 5.4 CD40

CD40 exists on the surface of both tumor and immune cells. It indirectly activates anti-tumor T cells by stimulating DCs to release IL-12. CD40 agonist reduces the density of the PDAC matrix, remodels macrophages, amplifies the function of DCs, and contributes to the proliferation and activation of T cells (196). Chemotherapy prior to the application of CD40 agonist can further ameliorate the therapeutic efficacy (197).

## 6 Summary and discussion

To sum up, most patients with PDAC missed the best operation time due to the difficulty of early diagnosis. Non-surgical treatments, such as chemotherapy and radiotherapy, have shown significant resistance against PDAC. Due to the high incidence rate of PDAC and the lack of effective treatment options, immunotherapy has emerged as a promising solution to its aggressive nature and poor prognosis. TME of PDAC has the rich and dense matrix, complex heterogeneous cell population, and ambiguous intercellular and intracellular soluble factor-dependent crosstalk mechanism. These facts make the “cold tumor” PDAC have irreversible immunosuppression. Although the current immunotherapy for PDAC is mostly focused on the preclinical stage and has failed to acquire exciting success, a deep understanding of the immunosuppressive mechanism of PDAC can facilitate the immunotherapy scheme to be better designed. This paper reviews PDAC, including its acidic microenvironment of hypoxia and nutrient deficiency, the complex composition of tumor cells, stromal cells, and immune cells in TME, multiple important signal pathways that are expected to become therapeutic targets, and the main immunotherapy strategies of PDAC. These components of the PDAC microenvironment communicate and interact with each other to form a significant immune suppression system and potential anti-tumor immune response. A comprehensive integration and introduction of the immunosuppressive properties to PDAC is conducive to their subsequent progress. Through promising immunotherapy and targeted therapy, transforming PDAC into “a hot tumor” is not illusory. However, the complex TME supports the immune escape of PDAC, and the conversion of preclinical studies of immunotherapy into clinical results still requires further exploration. Future immunotherapies will be optimized, such as new immunotherapeutic targets identified in the preclinical setting, as well as improvements in the durability and affordability of adoptive cell therapy. Anti-tumor immune cells will also be investigated for ways to promote their activation and enhanced efficacy. Immunotherapy has both accuracy and limitations. Under the initiative of individualized medicine, it is a huge challenge to successfully screen patients who have more opportunities to benefit from certain immunotherapy methods. An alternative train of thought is to expand the beneficiaries of immunotherapy. The exploration of current immunotherapy of PDAC shows that combination therapy may be more promising than any single therapy to benefit pancreatic cancer patients. For instance, neoadjuvant chemotherapy can improve the microenvironment of PDAC and expedite the effectiveness of immunotherapy (198). Optimal timing, dosing and selection of combination therapies may become major challenges. To comprehensively address the TME of PDAC, it may be necessary to seek more systematic and comprehensive treatment options to guide future development. Additionally, it is important to consider the adverse reactions and cost-effectiveness of these innovative treatment approaches.

In general, the immunosuppressive complex network of PDAC and immunotherapy with patient benefit prospects have the value of further research. Immunotherapy to alleviate immunosuppression may be an effective way to reverse the low survival rate of PDAC.

## Author contributions

SG: Writing – review & editing, Writing – original draft, Methodology. ZW: Writing – review & editing, Writing – original draft, Supervision, Methodology, Data curation.

## Glossary

**Table:**

PDAC	pancreatic ductal adenocarcinoma
TME	tumor microenvironment
ICB	immunocheckpoint block
ICI	immunocheckpoint inhibitors
ECM	Extracellular matrix
PSCs	pancreatic stellate cells
CAFs	cancer-associated fibroblasts
PanIN	pancreatic intraepithelial neoplasia
VEGF	vascular endothelial growth factor
TAMs	tumor-associated macrophages
IFP	interstitial fluid pressure
TGF-β	transforming growth factor-β
FAK	focal adhesion kinase
PEGPH20	PEGylated hyaluronidase
PMN	pre-metastatic Niche
Tregs	regulatory T cells
VEGFA	vascular endothelial growth factor A
TNF-α	tumor necrosis factor-α
αSMA	α-smooth muscle actin
myCAFs	myofibroblastic CAFs
iCAFs	inflammatory CAFs
apCAFs	antigen-presenting CAFs
FAP	fibroblast activation protein A
IL-1α	Interleukin-1α
LIF	leukemia inhibitory factor
Hh	hedgehog
ATRA	all trans-retinoic acid
CCR2	CXC chemokine receptor 2
CCL2	chemokine ligand 2
iNOS	inducible nitric oxide synthase
ARG- 1	arginase-1
IL-12	Interleukin-12
IL-10	Interleukin-10
IFN-γ	Interferon-γ
TLR	Toll-like receptors
ANG	angiopoietin ligand
TNF-α	tumor necrosis factor-α
ROS	Robot Operating System
PI3Kγ	phosphatidylinositol 3kinase γ
TANs	tumor-associated neutrophils
HGF	hepatocyte growth factor
MMP	matrix metalloprotinase
NGAL	neutrophil gelatinase-associated lipocalin
IL-1β	Interleukin-1β
NRF2	NF-E2 p45-related factor 2
E-cad	E-cadherin
NETs	Neutrophil extracellular traps
PADI4	peptide arginine deimidase 4
NLR	Neutrophil/lymphocyte ratio
MDSCs	myeloid suppressor cells
GM-CSF	granulocyte macrophage colony stimulating factor
G-CSF	granulocyte colony stimulating factor
S1P	sphingosine-1-phosphate
NOX2	NADPH oxidase 2
NO	nitric oxide
IDO	indoleamine 2, 3-dioxygenase
CTL	cytotoxic lymphocytes
TLS	tertiary lymphoid structure
CAR	chimeric antigen receptor
DCs	dendritic cells
APCs	antigen-presenting cells
PDGF-β	platelet-derived growth factor-β
LOXL2	Recombinant Lysyl Oxidase Like Protein 2
BTK	Bruton tyrosine kinase
MCs	mast cells
CSCs	cancer stem cells
TCF7	transcription factor 7
GOT2	glutamic- oxaloacetic transaminase 2
VDR	vitamin D receptor
SHIP-1	SH-2 containing inositol 5'polyphosphatase 1
UQCRC1	Ubiquinol-cytochrome c reductase core protein 1
IRAK4	Interleukin 1 Receptor Associated Kinase 4
LIPH	lipase H
DCLK1	Doublecortin Like Kinase 1
MSCs	mesenchymal stem cells
EMT	epithelial-to-mesenchymal transition
PTEN	Phosphatase and tensin homolog
Wif1	WNT inhibitory factor 1
GPC1	Glypican1
EVs	extracellular vesicles

## References

[1] Cronin KA Scott S Firth AU Sung H Henley SJ Sherman RL. Annual report to the nation on the status of cancer, part 1: National cancer statistics. Cancer. (2022) 128:4251–84. doi: 10.1002/cncr.34479 PMC10092838 36301149

[2] Ilic M Ilic I. Epidemiology of pancreatic cancer. World J Gastroenterol. (2016) 22:9694–705. doi: 10.3748/wjg.v22.i44.9694 PMC5124974 27956793

[3] Puleo F Nicolle R Blum Y Cros J Marisa L Demetter P. Stratification of pancreatic ductal adenocarcinomas based on tumor and microenvironment features. Gastroenterology. (2018) 155:1999–2013. doi: 10.1053/j.gastro.2018.08.033 30165049

[4] Advancing on pancreatic cancer. Nat Rev Gastroenterol Hepatol. (2021) 18:447. doi: 10.1038/s41575-021-00479-5 34188210

[5] Mizrahi JD Surana R Valle JW Shroff RT. Pancreatic cancer. Lancet. (2020) 395:2008–20. doi: 10.1016/S0140-6736(20)30974-0 32593337

[6] Mayo SC Gilson MM Herman JM Cameron JL Nathan H Edil BH. Management of patients with pancreatic adenocarcinoma: national trends in patient selection, operative management, and use of adjuvant therapy. J Am Coll Surg. (2012) 214:33–45. doi: 10.1016/j.jamcollsurg.2011.09.022 22055585 PMC3578342

[7] Barreto JN McCullough KB Ice LL Smith JA. Antineoplastic agents and the associated myelosuppressive effects: a review. J Pharm Pract. (2014) 27:440–6. doi: 10.1177/0897190014546108 25147158

[8] Zhu YH Zheng JH Jia QY Duan ZH Yao HF Yang J. Immunosuppression, immune escape, and immunotherapy in pancreatic cancer: focused on the tumor microenvironment. Cell Oncol (Dordr). (2023) 46:17–48. doi: 10.1007/s13402-022-00741-1 36367669 PMC12974690

[9] Zhao P Li L Jiang X Li Q. Mismatch repair deficiency/microsatellite instability-high as a predictor for anti-PD-1/PD-L1 immunotherapy efficacy. J Hematol Oncol. (2019) 12:54. doi: 10.1186/s13045-019-0738-1 31151482 PMC6544911

[10] Hessmann E Buchholz SM Demir IE Singh SK Gress TM Ellenrieder V. Microenvironmental determinants of pancreatic cancer. Physiol Rev. (2020) 100:1707–51. doi: 10.1152/physrev.00042.2019 32297835

[11] Agostini A Orlacchio A Carbone C Guerriero I. Understanding tricky cellular and molecular interactions in pancreatic tumor microenvironment: new food for thought. Front Immunol. (2022) 13:876291. doi: 10.3389/fimmu.2022.876291 35711414 PMC9193393

[12] Bilotta MT Antignani A Fitzgerald DJ. Managing the TME to improve the efficacy of cancer therapy. Front Immunol. (2022) 13:954992. doi: 10.3389/fimmu.2022.954992 36341428 PMC9630343

[13] Chan-Seng-Yue M Kim JC Wilson GW Ng K Figueroa EF O’Kane GM. Transcription phenotypes of pancreatic cancer are driven by genomic events during tumor evolution. Nat Genet. (2020) 52:231–40. doi: 10.1038/s41588-019-0566-9 31932696

[14] Velasco RM García AG Sánchez PJ Sellart IM Sánchez-Arévalo Lobo VJ. Tumour microenvironment and heterotypic interactions in pancreatic cancer. J Physiol Biochem. (2023) 79:179–92. doi: 10.1007/s13105-022-00875-8 35102531

[15] Ren B Cui M Yang G Wang H Feng M You L. Tumor microenvironment participates in metastasis of pancreatic cancer. Mol Cancer. (2018) 17:108. doi: 10.1186/s12943-018-0858-1 30060755 PMC6065152

[16] Helms E Onate MK Sherman MH. Fibroblast heterogeneity in the pancreatic tumor microenvironment. Cancer Discovery. (2020) 10:648–56. doi: 10.1158/2159-8290.CD-19-1353 PMC8261791 32014869

[17] Hosein AN Brekken RA Maitra A. Pancreatic cancer stroma: an update on therapeutic targeting strategies. Nat Rev Gastroenterol Hepatol. (2020) 17:487–505. doi: 10.1038/s41575-020-0300-1 32393771 PMC8284850

[18] Zhan HX Zhou B Cheng YG Xu JW Wang L Zhang GY. Crosstalk between stromal cells and cancer cells in pancreatic cancer: New insights into stromal biology. Cancer Lett. (2017) 392:83–93. doi: 10.1016/j.canlet.2017.01.041 28189533

[19] Murakami T Hiroshima Y Matsuyama R Homma Y Hoffman RM Endo I. Role of the tumor microenvironment in pancreatic cancer. Ann Gastroenterol Surg. (2019) 3:130–7. doi: 10.1002/ags3.12225 PMC6422798 30923782

[20] Gkretsi V Stylianou A Papageorgis P Polydorou C Stylianopoulos T. Remodeling components of the tumor microenvironment to enhance cancer therapy. Front Oncol. (2015) 5:214. doi: 10.3389/fonc.2015.00214 26528429 PMC4604307

[21] Mason BN Starchenko A Williams RM Bonassar LJ Reinhart-King CA. Tuning three-dimensional collagen matrix stiffness independently of collagen concentration modulates endothelial cell behavior. Acta Biomater. (2013) 9:4635–44. doi: 10.1016/j.actbio.2012.08.007 PMC3508162 22902816

[22] Tian C Öhlund D Rickelt S Lidström T Huang Y Hao L. Cancer cell-derived matrisome proteins promote metastasis in pancreatic ductal adenocarcinoma. Cancer Res. (2020) 80:1461–74. doi: 10.1158/0008-5472.CAN-19-2578 PMC7127978 32029550

[23] Schizas D Charalampakis N Kole C Economopoulou P Koustas E Gkotsis E. Immunotherapy for pancreatic cancer: A 2020 update. Cancer Treat Rev. (2020) 86:102016. doi: 10.1016/j.ctrv.2020.102016 32247999

[24] Nevala-Plagemann C Hidalgo M Garrido-Laguna I. From state-of-the-art treatments to novel therapies for advanced-stage pancreatic cancer. Nat Rev Clin Oncol. (2020) 17:108–23. doi: 10.1038/s41571-019-0281-6 31705130

[25] Jacobetz MA Chan DS Neesse A Bapiro TE Cook N Frese KK. Hyaluronan impairs vascular function and drug delivery in a mouse model of pancreatic cancer. Gut. (2013) 62:112–20. doi: 10.1136/gutjnl-2012-302529 PMC3551211 22466618

[26] Bejarano L Jordāo MJC Joyce JA. Therapeutic targeting of the tumor microenvironment. Cancer Discovery. (2021) 11:933–59. doi: 10.1158/2159-8290.CD-20-1808 33811125

[27] Hamidi H Ivaska J. Every step of the way: integrins in cancer progression and metastasis. Nat Rev Cancer. (2018) 18:533–48. doi: 10.1038/s41568-018-0038-z PMC6629548 30002479

[28] Zeltz C Primac I Erusappan P Alam J Noel A Gullberg D. Cancer-associated fibroblasts in desmoplastic tumors: emerging role of integrins. Semin Cancer Biol. (2020) 62:166–81. doi: 10.1016/j.semcancer.2019.08.004 31415910

[29] Argentiero A Calabrese A Solimando AG Notaristefano A Panarelli MMG Brunetti O. Bone metastasis as primary presentation of pancreatic ductal adenocarcinoma: A case report and literature review. Clin Case Rep. (2019) 7:1972–6. doi: 10.1002/ccr3.2412 PMC6787833 31624620

[30] Altorki NK Markowitz GJ Gao D Port JL Saxena A Stiles B. The lung microenvironment: an important regulator of tumour growth and metastasis. Nat Rev Cancer. (2019) 19:9–31. doi: 10.1038/s41568-018-0081-9 30532012 PMC6749995

[31] Peinado H Zhang H Matei IR Costa-Silva B Hoshino A Rodrigues G. Pre-metastatic niches: organ-specific homes for metastases. Nat Rev Cancer. (2017) 17:302–17. doi: 10.1038/nrc.2017.6 28303905

[32] Giraldo NA Sanchez-Salas R Peske JD Vano Y Becht E Petitprez F. The clinical role of the TME in solid cance. Br J Cancer. (2019) 120:45–53. doi: 10.1038/s41416-018-0327-z 30413828 PMC6325164

[33] Jiang H Torphy RJ Steiger K Hongo H Ritchie AJ Kriegsmann M. Pancreatic ductal adenocarcinoma progression is restrained by stromal matrix. J Clin Invest. (2020) 130:4704–9. doi: 10.1172/JCI136760 PMC7456216 32749238

[34] Ino Y Yamazaki-Itoh R Shimada K Iwasaki M Kosuge T Kanai Y. Immune cell infiltration as an indicator of the immune microenvironment of pancreatic cancer. Br J Cancer. (2013) 108:914–23. doi: 10.1038/bjc.2013.32 PMC3590668 23385730

[35] Zhang Z Zhang H Shi L Wang D Tang D. Heterogeneous cancer-associated fibroblasts: A new perspective for understanding immunosuppression in pancreatic cancer. Immunology. (2022) 167:1–14. doi: 10.1111/imm.13496 35569095

[36] Özdemir BC Pentcheva-Hoang T Carstens JL Zheng X Wu CC Simpson TR. Depletion of carcinoma-associated fibroblasts and fibrosis induces immunosuppression and accelerates pancreas cancer with reduced survival. Cancer Cell. (2014) 25:719–34. doi: 10.1016/j.ccr.2014.04.005 PMC4180632 24856586

[37] Öhlund D Handly-Santana A Biffi G Elyada E Almeida AS Ponz-Sarvise M. Distinct populations of inflammatory fibroblasts and myofibroblasts in pancreatic cancer. J Exp Med. (2017) 214:579–96. doi: 10.1084/jem.20162024 PMC5339682 28232471

[38] Sahai E Astsaturov I Cukierman E DeNardo DG Egeblad M Evans RM. A framework for advancing our understanding of cancer-associated fibroblasts. Nat Rev Cancer. (2020) 20:174–86. doi: 10.1038/s41568-019-0238-1 PMC7046529 31980749

[39] Elyada E Bolisetty M Laise P Flynn WF Courtois ET Burkhart RA. Cross-species single-cell analysis of pancreatic ductal adenocarcinoma reveals antigen-presenting cancer-associated fibroblasts. Cancer Discovery. (2019) 9:1102–23. doi: 10.1158/2159-8290.CD-19-0094 PMC6727976 31197017

[40] Kerdidani D Aerakis E Verrou KM Angelidis I Douka K Maniou MA. Lung tumor MHCII immunity depends on in *situ* antigen presentation by fibroblasts. J Exp Med. (2022) 219:e20210815. doi: 10.1084/jem.20210815 35029648 PMC8764966

[41] Cheng CS Yang PW Sun Y Song SL Chen Z. Fibroblast activation protein-based theranostics in pancreatic cancer. Front Oncol. (2022) 12:969731. doi: 10.3389/fonc.2022.969731 36263225 PMC9574192

[42] Biffi G Oni TE Spielman B Hao Y Elyada E Park Y. IL1-induced JAK/STAT signaling is antagonized by TGFβ to shape CAF heterogeneity in pancreatic ductal adenocarcinoma. Cancer Discovery. (2019) 9:282–301. doi: 10.1158/2159-8290.CD-18-0710 30366930 PMC6368881

[43] Steele NG Biffi G Kemp SB Zhang Y Drouillard D Syu L. Inhibition of hedgehog signaling alters fibroblast composition in pancreatic cancer. Clin Cancer Res. (2021) 27:2023–37. doi: 10.1158/1078-0432.CCR-20-3715 PMC8026631 33495315

[44] Awaji M Saxena S Wu L Prajapati DR Purohit A Varney ML. CXCR2 signaling promotes secretory cancer-associated fibroblasts in pancreatic ductal adenocarcinoma. FASEB J. (2020) 34:9405–18. doi: 10.1096/fj.201902990R PMC7501205 32453916

[45] Vennin C Mélénec P Rouet R Nobis M Cazet AS Murphy KJ. CAF hierarchy driven by pancreatic cancer cell p53-status creates a pro-metastatic and chemoresistant environment via perlecan. Nat Commun. (2019) 10:3637. doi: 10.1038/s41467-019-10968-6 31406163 PMC6691013

[46] Shinkawa T Ohuchida K Nakamura M. Heterogeneity of cancer-associated fibroblasts and the tumor immune microenvironment in pancreatic cancer. Cancers (Basel). (2022) 14:3994. doi: 10.3390/cancers14163994 36010986 PMC9406547

[47] Kocher HM Basu B Froeling FEM Sarker D Slater S Carlin D. Phase I clinical trial repurposing all-trans retinoic acid as a stromal targeting agent for pancreatic cancer. Nat Commun. (2020) 11:4841. doi: 10.1038/s41467-020-18636-w 32973176 PMC7518421

[48] Zhu Y Herndon JM Sojka DK Kim KW Knolhoff BL Zuo C. Tissue-resident macrophages in pancreatic ductal adenocarcinoma originate from embryonic hematopoiesis and promote tumor progression. Immunity. (2017) 47:597. doi: 10.1016/j.immuni.2017.08.018 28930665 PMC5664180

[49] Hu H Hang J-J Han T Zhuo M Jiao F Wang LW. The M2 phenotype of tumor-associated macrophages in the stroma confers a poor prognosis in pancreatic cancer. Tumour Biol. (2016) 37:8657–64. doi: 10.1007/s13277-015-4741-z 26738860

[50] Pratt HG Steinberger KJ Mihalik NE Ott S Whalley T Szomolay B. Macrophage and neutrophil interactions in the pancreatic tumor microenvironment drive the pathogenesis of pancreatic cancer. Cancers (Basel). (2021) 14:194. doi: 10.3390/cancers14010194 35008355 PMC8750413

[51] Yang M Li J Gu P Fan X. The application of nanoparticles in cancer immunotherapy: Targeting tumor microenvironment. Bioact Mater. (2020) 6:1973–87. doi: 10.1016/j.bioactmat.2020.12.010 PMC7773537 33426371

[52] Gabrilovich DI Ostrand-Rosenberg S Bronte V. Coordinated regulation of myeloid cells by tumours. Nat Rev Immunol. (2012) 12:253–68. doi: 10.1038/nri3175 PMC3587148 22437938

[53] Ruffell B Chang-Strachan D Chan V Rosenbusch A Ho CM Pryer N. Macrophage IL-10 blocks CD8^+^ T cell-dependent responses to chemotherapy by suppressing IL-12 expression in intratumoral dendritic cells. Cancer Cell. (2014) 26:623–37. doi: 10.1016/j.ccell.2014.09.006 PMC4254570 25446896

[54] Ullman NA Burchard PR Dunne RF Linehan DC. Immunologic strategies in pancreatic cancer: making *cold* tumors *hot* . J Clin Oncol. (2022) 40:2789–805. doi: 10.1200/JCO.21.02616 PMC9390820 35839445

[55] Zhang J Li R Huang S. The immunoregulation effect of tumor microenvironment in pancreatic ductal adenocarcinoma. Front Oncol. (2022) 12:951019. doi: 10.3389/fonc.2022.951019 35965504 PMC9365986

[56] Akwii RG Sajib MS Zahra FT Mikelis CM. Role of angiopoietin-2 in vascular physiology and pathophysiology. Cells. (2019) 8:471. doi: 10.3390/cells8050471 31108880 PMC6562915

[57] Roiniotis J Dinh H Masendycz P Turner A Elsegood CL Scholz GM. Hypoxia prolongs monocyte/macrophage survival and enhanced glycolysis is associated with their maturation under aerobic conditions. J Immunol. (2009) 182:7974–81. doi: 10.4049/jimmunol.0804216 19494322

[58] Martinez FO Gordon S. The M1 and M2 paradigm of macrophage activation: time for reassessment. F1000Prime Rep. (2014) 6:13. doi: 10.12703/P6-13 24669294 PMC3944738

[59] Elinav E Nowarski R Thaiss CA Hu B Jin C Flavell RA. Inflammation-induced cancer: crosstalk between tumours, immune cells and microorganisms. Nat Rev Cancer. (2013) 13:759–71. doi: 10.1038/nrc3611 24154716

[60] Dou A Fang J. Heterogeneous myeloid cells in tumors. Cancers (Basel). (2021) 13:3772. doi: 10.3390/cancers13153772 34359674 PMC8345207

[61] Jiang W Li X Xiang C Zhou W. Neutrophils in pancreatic cancer: Potential therapeutic targets. Front Oncol. (2022) 12:1025805. doi: 10.3389/fonc.2022.1025805 36324574 PMC9618950

[62] Fridlender ZG Sun J Kim S Kapoor V Cheng G Ling L. Polarization of tumor-associated neutrophil phenotype by TGF-beta: "N1" versus "N2" TAN. Cancer Cell. (2009) 16:183–94. doi: 10.1016/j.ccr.2009.06.017 PMC2754404 19732719

[63] Jaillon S Ponzetta A Di Mitri D Santoni A Bonecchi R Mantovani A. Neutrophil diversity and plasticity in tumour progression and therapy. Nat Rev Cancer. (2020) 20:485–503. doi: 10.1038/s41568-020-0281-y 32694624

[64] Zhou SL Zhou ZJ Hu ZQ Huang XW Wang Z Chen EB. Tumor-associated neutrophils recruit macrophages and T-regulatory cells to promote progression of hepatocellular carcinoma and resistance to sorafenib. Gastroenterology. (2016) 150:1646–1658. e17. doi: 10.1053/j.gastro.2016.02.040 26924089

[65] Crescenzi E Leonardi A Pacifico F. NGAL as a potential target in tumor microenvironment. Int J Mol Sci. (2021) 22:12333. doi: 10.3390/ijms222212333 34830212 PMC8623964

[66] Incio J Liu H Suboj P Chin SM Chen IX Pinter M. Obesity-induced inflammation and desmoplasia promote pancreatic cancer progression and resistance to chemotherapy. Cancer Discovery. (2016) 6:852–69. doi: 10.1158/2159-8290.CD-15-1177 PMC4972679 27246539

[67] Wang X Yuan X Su Y Hu J Ji Q Fu S. Targeting purinergic receptor P2RX1 modulates intestinal microbiota and alleviates inflammation in colitis. Front Immunol. (2021) 12:696766. doi: 10.3389/fimmu.2021.696766 34354708 PMC8329583

[68] Wang X Hu LP Qin WT Yang Q Chen DY Li Q. Identification of a subset of immunosuppressive P2RX1-negative neutrophils in pancreatic cancer liver metastasis. Nat Commun. (2021) 12:174. doi: 10.1038/s41467-020-20447-y 33420030 PMC7794439

[69] Cuadrado A Rojo AI Wells G Hayes JD Cousin SP Rumsey WL. Therapeutic targeting of the NRF2 and KEAP1 partnership in chronic diseases. Nat Rev Drug Discovery. (2019) 18:295–317. doi: 10.1038/s41573-018-0008-x 30610225

[70] Kajioka H Kagawa S Ito A Yoshimoto M Sakamoto S Kikuchi S. Targeting neutrophil extracellular traps with thrombomodulin prevents pancreatic cancer metastasis. Cancer Lett. (2021) 497:1–13. doi: 10.1016/j.canlet.2020.10.015 33065249

[71] Chao T Furth EE Vonderheide RH. CXCR2-dependent accumulation of tumor-associated neutrophils regulates T-cell immunity in pancreatic ductal adenocarcinoma. Cancer Immunol Res. (2016) 4:968–82. doi: 10.1158/2326-6066.CIR-16-0188 PMC5110270 27737879

[72] Zhang Y Chandra V Riquelme Sanchez E Dutta P Quesada PR Rakoski A. Interleukin-17-induced neutrophil extracellular traps mediate resistance to checkpoint blockade in pancreatic cancer. J Exp Med. (2020) 217:e20190354. doi: 10.1084/jem.20190354 32860704 PMC7953739

[73] Poto R Cristinziano L Modestino L de Paulis A Marone G Loffredo S. Neutrophil extracellular traps, angiogenesis and cancer. Biomedicines. (2022) 10:431. doi: 10.3390/biomedicines10020431 35203640 PMC8962440

[74] Boone BA Orlichenko L Schapiro NE Loughran P Gianfrate GC Ellis JT. The receptor for advanced glycation end products (RAGE) enhances autophagy and neutrophil extracellular traps in pancreatic cancer. Cancer Gene Ther. (2015) 22:326–34. doi: 10.1038/cgt.2015.21 PMC4470814 25908451

[75] Jin W Xu HX Zhang SR Li H Wang WQ Gao HL. Tumor-infiltrating NETs predict postsurgical survival in patients with pancreatic ductal adenocarcinoma. Ann Surg Oncol. (2019) 26:635–43. doi: 10.1245/s10434-018-6941-4 30374923

[76] Cristinziano L Modestino L Antonelli A Marone G Simon HU Varricchi G. Neutrophil extracellular traps in cancer. Semin Cancer Biol. (2022) 79:91–104. doi: 10.1016/j.semcancer.2021.07.011 34280576

[77] Nywening TM Belt BA Cullinan DR Panni RZ Han BJ Sanford DE. Targeting both tumour-associated CXCR2^+^ neutrophils and CCR2^+^ macrophages disrupts myeloid recruitment and improves chemotherapeutic responses in pancreatic ductal adenocarcinoma. Gut. (2018) 67:1112–23. doi: 10.1136/gutjnl2017-313738 PMC5969359 29196437

[78] O'Hara MH Messersmith W Kindler H Zhang W Pitou C Szpurka AM. Safety and pharmacokinetics of CXCR4 peptide antagonist, LY2510924, in combination with durvalumab in advanced refractory solid tumors. J Pancreat Cancer. (2020) 6:21–31. doi: 10.1089/pancan.2019.0018 32219196 PMC7097682

[79] Melisi D Oh D-Y Hollebecque A Calvo E Varghese A Borazanci E. Safety and activity of the TGFβ receptor I kinase inhibitor galunisertib plus the anti-PD-L1 antibody durvalumab in metastatic pancreatic cancer. J Immunother Cancer. (2021) 9:e002068. doi: 10.1136/jitc-2020-002068 33688022 PMC7944986

[80] McAllister F Bailey JM Alsina J Nirschl CJ Sharma R Fan H. Oncogenic Kras activates a hematopoietic-to-epithelial IL-17 signaling axis in preinvasive pancreatic neoplasia. Cancer Cell. (2014) 25:621–37. doi: 10.1016/j.ccr.2014.03.014 PMC4072043 24823639

[81] Templeton AJ McNamara MG Šeruga B Vera-Badillo FE Aneja P Ocaña A. Prognostic role of neutrophil-to-lymphocyte ratio in solid tumors: a systematic review and meta-analysis. J Natl Cancer Inst. (2014) 106:dju124. doi: 10.1093/jnci/dju124 24875653

[82] Wu WC Sun HW Chen J OuYang HY Yu XJ Chen HT. Immunosuppressive immature myeloid cell generation is controlled by glutamine metabolism in human cancer. Cancer Immunol Res. (2019) 7:1605–18. doi: 10.1158/2326-6066.CIR-18-0902 31387898

[83] Kemp SB di Magliano M Crawford HC. Myeloid cell mediated immune suppression in pancreatic cancer. Cell Mol Gastroenterol Hepatol. (2021) 12:1531–42. doi: 10.1016/j.jcmgh.2021.07.006 PMC8529393 34303882

[84] Li B-H Garstka MA Li Z-F. Chemokines and their receptors promoting the recruitment of myeloid-derived suppressor cells into the tumor. Mol Immunol. (2020) 117:201–15. doi: 10.1016/j.molimm.2019.11.014 31835202

[85] Li W Zhang X Chen Y Xie Y Liu J Feng Q. G-CSF is a key modulator of MDSC and could be a potential therapeutic target in colitis-associated colorectal cancers. Protein Cell. (2016) 7:130–40. doi: 10.1007/s13238-015-0237-2 PMC4742385 26797765

[86] Li Y Wang J Wang H Zhang S Wei Y Liu S. The interplay between inflammation and stromal components in pancreatic cancer. Front Immunol. (2022) 13:850093. doi: 10.3389/fimmu.2022.850093 35493517 PMC9046560

[87] Chen JY Lai YS Chu PY Chan S-H Wang LH Hung WC. Cancer-derived VEGF-C increases chemokine production in lymphatic endothelial cells to promote CXCR2-dependent cancer invasion and MDSC recruitment. Cancers (Basel). (2019) 11:1120. doi: 10.3390/cancers11081120 31390756 PMC6721484

[88] Grzywa TM Sosnowska A Matryba P Rydzynska Z Jasinski M Nowis D. Myeloid cell-derived arginase in cancer immune response. Front Immunol. (2020) 11:938. doi: 10.3389/fimmu.2020.00938 32499785 PMC7242730

[89] Pinton L Solito S Damuzzo V Francescato S Pozzuoli A Berizzi A. Activated T cells sustain myeloid-derived suppressor cell-mediated immune suppression. Oncotarget. (2016) 7:1168–84. doi: 10.18632/oncotarget.6662 PMC4811451 26700461

[90] Jacob A Prekeris R. The regulation of MMP targeting to invadopodia during cancer metastasis. Front Cell Dev Biol. (2015) 3:4. doi: 10.3389/fcell.2015.00004 25699257 PMC4313772

[91] Heine A Flores C Gevensleben H Diehl L Heikenwalder M Ringelhan M. Targeting myeloid derived suppressor cells with all-trans retinoic acid is highly time-dependent in therapeutic tumor vaccination. Oncoimmunology. (2017) 6:e1338995. doi: 10.1080/2162402X.2017.1338995 28920004 PMC5593699

[92] Porta C Consonni FM Morlacchi S Sangaletti S Bleve A Totaro MG. Tumor-derived prostaglandin E2 promotes p50 NF-κB-dependent differentiation of monocytic MDSCs. Cancer Res. (2020) 80:2874–88. doi: 10.1158/0008-5472.CAN-19-2843 32265223

[93] Choueiry F Torok M Shakya R Agrawal K Deems A Benner B. CD200 promotes immunosuppression in the pancreatic tumor microenvironment. J Immunother Cancer. (2020) 8:e000189. doi: 10.1136/jitc-2019-000189 32581043 PMC7312341

[94] Yang X Lu Y Hang J Zhang J Zhang T Huo Y. Lactate-modulated immunosuppression of myeloid-derived suppressor cells contributes to the radioresistance of pancreatic cancer. Cancer Immunol Res. (2020) 8:1440–51. doi: 10.1158/2326-6066.CIR-20-0111 32917658

[95] Liu Q Wu H Li Y Zhang R Kleeff J Zhang X. Combined blockade of TGf-β1 and GM-CSF improves chemotherapeutic effects for pancreatic cancer by modulating tumor microenvironment. Cancer Immunol Immunother. (2020) 69:1477–92. doi: 10.1007/s00262-020-02542-7 PMC11027661 32285172

[96] Gajewski TF Schreiber H Fu YX. Innate and adaptive immune cells in the tumor microenvironment. Nat Immunol. (2013) 14:1014–22. doi: 10.1038/ni.2703 PMC4118725 24048123

[97] Sun H Zhang B Li H. The roles of frequently mutated genes of pancreatic cancer in regulation of tumor microenvironmentt. Technol Cancer Res Treat. (2020) 19:1533033820920969. doi: 10.1177/1533033820920969 32372692 PMC7225789

[98] Stone ML Beatty GL. Cellular determinants and therapeutic implications of inflammation in pancreatic cancer. Pharmacol Ther. (2019) 201:202–13. doi: 10.1016/j.pharmthera.2019.05.012 PMC6708742 31158393

[99] Uzunparmak B Sahin IH. Pancreatic cancer microenvironment: a current dilemma. Clin Transl Med. (2019) 8:2. doi: 10.1186/s40169-019-0221-1 30645701 PMC6333596

[100] Johnson BA 3rd Yarchoan M Lee V Laheru DA Jaffee EM. Strategies for increasing pancreatic tumor immunogenicity. Clin Cancer Res. (2017) 23:1656–69. doi: 10.1158/1078-0432.CCR-16-2318 PMC5466881 28373364

[101] Goulart MR Stasinos K Fincham REA Delvecchio FR Kocher HM. T cells in pancreatic cancer stroma. World J Gastroenterol. (2021) 27:7956–68. doi: 10.3748/wjg.v27.i46.7956 PMC8678814 35046623

[102] Hiraoka N Ino Y Yamazaki-Itoh R Kanai Y Kosuge T Shimada K. Intratumoral tertiary lymphoid organ is a favourable prognosticator in patients with pancreatic cancer. Br J Cancer. (2015) 112:1782–90. doi: 10.1038/bjc.2015.145 PMC4647237 25942397

[103] Yarchoan M Johnson BA 3rd Lutz ER Laheru DA Jaffee EM. Targeting neoantigens to augment antitumour immunity. Nat Rev Cancer. (2017) 17:209–22. doi: 10.1038/nrc.2016.154 PMC5575801 28233802

[104] Tesfaye AA Kamgar M Azmi A Philip PA. The evolution into personalized therapies in pancreatic ductal adenocarcinoma: challenges and opportunities. Expert Rev Anticancer Ther. (2018) 18:131–48. doi: 10.1080/14737140.2018.1417844 PMC6121777 29254387

[105] Ho TTB Nasti A Seki A Komura T Inui H Kozaka T. Combination of gemcitabine and anti-PD-1 antibody enhances the anticancer effect of M1 macrophages and the Th1 response in a murine model of pancreatic cancer liver metastasis. J Immunother Cancer. (2020) 8:e001367. doi: 10.1136/jitc-2020-001367 33188035 PMC7668383

[106] Alam A Levanduski E Denz P Villavicencio HS Bhatta M Alhorebi L. Fungal mycobiome drives IL-33 secretion and type 2 immunity in pancreatic cancer. Cancer Cell. (2022) 40:153–167.e11. doi: 10.1016/j.ccell.2022.01.003 35120601 PMC8847236

[107] Jang J-E Hajdu CH Liot C Miller G Dustin ML Bar-Sagi D. Crosstalk between regulatory T cells and tumor-associated dendritic cells negates anti-tumor immunity in pancreatic cancer. Cell Rep. (2017) 20:558–71. doi: 10.1016/j.celrep.2017.06.062 PMC5649374 28723561

[108] Schietinger A Greenberg PD. Tolerance and exhaustion: defining mechanisms of T cell dysfunction. Trends Immunol. (2014) 35:51–60. doi: 10.1016/j.it.2013.10.001 24210163 PMC3946600

[109] Seidel JA Otsuka A Kabashima K. Anti-PD-1 and anti-CTLA-4 therapies in cancer: mechanisms of action, efficacy, and limitations. Front Oncol. (2018) 8:86. doi: 10.3389/fonc.2018.00086 29644214 PMC5883082

[110] Saka D Gökalp M Piyade B Cevik NC Arik Sever E Unutmaz D. Mechanisms of T-cell exhaustion in pancreatic cancer. Cancers (Basel). (2020) 12:2274. doi: 10.3390/cancers12082274 32823814 PMC7464444

[111] Stromnes IM Hulbert A Pierce RH Greenberg PD Hingorani SR. T-cell localization, activation, and clonal expansion in human pancreatic ductal adenocarcinoma. Cancer Immunol Res. (2017) 5:978–91. doi: 10.1158/2326-6066.CIR-16-0322 PMC5802342 29066497

[112] Aida K Miyakawa R Suzuki K Narumi K Udagawa T Yamamoto Y. Suppression of Tregs by anti-glucocorticoid induced TNF receptor antibody enhances the antitumor immunity of interferon-α gene therapy for pancreatic cancer. Cancer Sci. (2014) 105:159–67. doi: 10.1111/cas.12332 PMC4317823 24289533

[113] Gardner A Ruffell B. Dendritic cells and cancer immunity. Trends Immunol. (2016) 37:855–65. doi: 10.1016/j.it.2016.09.006 PMC5135568 27793569

[114] Böttcher JP Reis e Sousa C. The role of type 1 conventional dendritic cells in cancer immunity. Trends Cancer. (2018) 4:784–92. doi: 10.1016/j.trecan.2018.09.001 PMC6207145 30352680

[115] Elia AR Cappello P Puppo M Fraone T Vanni C Eva A. Human dendritic cells differentiated in hypoxia down-modulate antigen uptake and change their chemokine expression profile. J Leukoc Biol. (2008) 84:1472–82. doi: 10.1189/jlb.0208082 18725395

[116] Barilla RM Diskin B Caso RC Lee KB Mohan N Buttar C. Specialized dendritic cells induce tumor-promoting IL-10^+^ IL-17^+^ FoxP3^neg^ regulatory CD4^+^ T cells in pancreatic carcinoma. Nat Commun. (2019) 10:1424. doi: 10.1038/s41467-019-09416-2 30926808 PMC6441038

[117] Yamamoto T Yanagimoto H Satoi S Toyokawa H Yamao J Kim S. Circulating myeloid dendritic cells as prognostic factors in patients with pancreatic cancer who have undergone surgical resection. J Surg Res. (2012) 173:299–308.doi: 10.1016/j.jss.2010.09.027 21195425

[118] Montfort A Pearce O Maniati E Vincent BG Bixby L Böhm S. A strong B-cell response is part of the immune landscape in human high-grade serous ovarian metastases. Clin Cancer Res. (2017) 23:250–62. doi: 10.1158/1078-0432.CCR-16-0081 PMC5928522 27354470

[119] Pylayeva-Gupta Y Das S Handler JS Hajdu CH Coffre M Koralov SB. IL35-producing B cells promote the development of pancreatic neoplasia. Cancer Discovery. (2016) 6:247–55. doi: 10.1158/2159-8290.CD-15-0843 PMC5709038 26715643

[120] Lee KE Spata M Bayne LJ Buza EL Durham AC Allman D. Hif1a deletion reveals pro-neoplastic function of B cells in pancreatic neoplasia. Cancer Discovery. (2016) 6:256–69. doi: 10.1158/2159-8290.CD-15-0822 PMC4783189 26715642

[121] Castino GF Cortese N Capretti G Serio S Di Caro G Mineri R. Spatial distribution of B cells predicts prognosis in human pancreatic adenocarcinoma. Oncoimmunology. (2015) 5:e1085147. doi: 10.1080/2162402X.2015.1085147 27141376 PMC4839336

[122] Minici C Testoni S Della-Torre E. B-lymphocytes in the pathophysiology of pancreatic adenocarcinoma. Front Immunol. (2022) 13:867902. doi: 10.3389/fimmu.2022.867902 35359944 PMC8963963

[123] Minici C Rigamonti E Lanzillotta M Monno A Rovati L Maehara T. B lymphocytes contribute to stromal reaction in pancreatic ductal adenocarcinoma. Oncoimmunology. (2020) 9:1794359. doi: 10.1080/2162402X.2020.1794359 32923157 PMC7458626

[124] Della-Torre E Rigamonti E Perugino C Baghai-Sain S Sun N Kaneko N. B lymphocytes directly contribute to tissue fibrosis in patients with IgG_4_-related disease. J Allergy Clin Immunol. (2020) 145:968–981.e14. doi: 10.1016/j.jaci.2019.07.004 31319101 PMC6960365

[125] Della-Torre E Feeney E Deshpande V Mattoo H Mahajan V Kulikova M. B-cell depletion attenuates serological biomarkers of fibrosis and myofibroblast activation in IgG4-related disease. Ann Rheum Dis. (2015) 74:2236–43. doi: 10.1136/annrheumdis-2014-205799 PMC4806785 25143523

[126] Lanzillotta M Della-Torre E Milani R Bozzolo E Bozzalla-Cassione E Rovati L. Increase of circulating memory B cells after glucocorticoid-induced remission identifies patients at risk of IgG4-related disease relapse. Arthritis Res Ther. (2018) 20:222. doi: 10.1186/s13075-018-1718-5 30285841 PMC6235221

[127] Yuen GJ Demissie E Pillai S. B lymphocytes and cancer: a love-hate relationship. Trends Cancer. (2016) 2:747–57. doi: 10.1016/j.trecan.2016.10.010 PMC5472356 28626801

[128] Zhao Y Shen M Feng Y He R Xu X Xie Y. Regulatory B cells induced by pancreatic cancer cell-derived interleukin-18 promote immune tolerance via the PD-1/PD-L1 pathway. Oncotarget. (2017) 9:14803–14. doi: 10.18632/oncotarget.22976 PMC5871079 29599908

[129] Tong DN Guan J Sun JH Zhao CY Chen SG Zhang ZY. Characterization of B cell-mediated PD-1/PD-L1 interaction in pancreatic cancer patients. Clin Exp Pharmacol Physiol. (2020) 47:1342–9. doi: 10.1111/1440-1681.13317 32248559

[130] Mirlekar B Michaud D Lee SJ Kren NP Harris C Greene K. B cell–derived IL35 drives STAT3-dependent CD8^+^ T-cell exclusion in pancreatic cancer. Cancer Immunol Res. (2020) 8:292–308. doi: 10.1158/2326-6066.CIR-19-0349 32024640 PMC7056532

[131] Das S Bar-Sagi D. BTK signaling drives CD1d^hi^CD5^+^ regulatory B-cell differentiation to promote pancreatic carcinogenesis. Oncogene. (2019) 38:3316–24. doi: 10.1038/s41388-018-0668-3 PMC6486434 30635655

[132] Spear S Candido JB McDermott JR Ghirelli C Maniati E Beers SA. Discrepancies in the tumor microenvironment of spontaneous and orthotopic murine models of pancreatic cancer uncover a new immunostimulatory phenotype for B cells. Front Immunol. (2019) 10:542. doi: 10.3389/fimmu.2019.00542 30972056 PMC6445859

[133] Remark R Alifano M Cremer I Lupo A Dieu-Nosjean MC Riquet M. Characteristics and clinical impacts of the immune environments in colorectal and renal cell carcinoma lung metastases: influence of tumor origin. Clin Cancer Res. (2013) 19:4079–91. doi: 10.1158/1078-0432.CCR-12-3847 23785047

[134] Gürlevik E Fleischmann-Mundt B Brooks J Demir IE Steiger K Ribback S. Administration of gemcitabine after pancreatic tumor resection in mice induces an antitumor immune response mediated by natural killer cells. Gastroenterology. (2016) 151:338–350.e7. doi: 10.1053/j.gastro.2016.05.004 27210037

[135] Muthalagu N Monteverde T Raffo-Iraolagoitia X Wiesheu R Whyte D Hedley A. Repression of the type I interferon pathway underlies MYC- and KRAS-dependent evasion of NK and B cells in pancreatic ductal adenocarcinoma. Cancer Discovery. (2020) 10:872–87. doi: 10.1158/2159-8290.CD-19-0620 PMC7611248 32200350

[136] Komi DEA Redegeld FA. Role of mast cells in shaping the tumor microenvironment. Clin Rev Allergy Immunol. (2020) 58:313–25. doi: 10.1007/s12016-019-08753-w PMC7244463 31256327

[137] Chang DZ Ma Y Ji B Wang H Deng D Liu Y. Mast cells in tumor microenvironment promotes the in *vivo* growth of pancreatic ductal adenocarcinoma. Clin Cancer Res. (2011) 17:7015–23. doi: 10.1158/1078-0432.CCR-11-0607 PMC4089502 21976550

[138] Krystel-Whittemore M Dileepan KN Wood JG. Mast cell: A multi-functional master cell. Front Immunol. (2016) 6:620. doi: 10.3389/fimmu.2015.00620 26779180 PMC4701915

[139] Ronca R Tamma R Coltrini D Ruggieri S Presta M Ribatti D. Fibroblast growth factor modulates mast cell recruitment in a murine model of prostate cancer. Oncotarget. (2017) 8:82583–92. doi: 10.18632/oncotarget.19773 PMC5669912 29137286

[140] Porcelli L Iacobazzi RM Di Fonte R Serratì S Intini A Solimando AG. CAFs and TGF-β Signaling activation by mast cells contribute to resistance to gemcitabine/nabpaclitaxel in pancreatic cancer. Cancers (Basel). (2019) 11:330. doi: 10.3390/cancers11030330 30866547 PMC6468868

[141] Eissmann MF Buchert M Ernst M. IL33 and mast cells-the key regulators of immune responses in gastrointestinal cancers? Front Immunol. (2020) 11:1389. doi: 10.3389/fimmu.2020.01389 32719677 PMC7350537

[142] Hashimoto O Shimizu K Semba S Chiba S Ku Y Yokozaki H. Hypoxia induces tumor aggressiveness and the expansion of CD133-positive cells in a hypoxia-inducible factor-1α-dependent manner in pancreatic cancer cells. Pathobiology. (2011) 78:181–92. doi: 10.1159/000325538 21778785

[143] Jaiswal KR Xin HW Anderson A Wiegand G Kim B Miller T. Comparative testing of various pancreatic cancer stem cells results in a novel class of pancreatic-cancer-initiating cells. Stem Cell Res. (2012) 9:249–60. doi: 10.1016/j.scr.2012.08.001 PMC3490042 22963768

[144] Su HT Weng CC Hsiao PJ Chen LH Kuo TL Chen YW. Stem cell marker nestin is critical for TGF-β1-mediated tumor progression in pancreatic cancerNestin mediates TGF-β1–induced EMT and tumor progression. Mol Cancer Res. (2013) 11:768–79. doi: 10.1158/1541-7786.MCR-12-0511 23552743

[145] Newsome RC Yang Y Jobin C. The microbiome, gastrointestinal cancer, and immunotherapy. J Gastroenterol Hepatol. (2022) 37:263–72. doi: 10.1111/jgh.15742 PMC9922516 34820895

[146] Nwosu ZC Pasca di Magliano M. GOT2: an unexpected mediator of immunosuppression in pancreatic cancer. Cancer Discovery. (2022) 12:2237–9. doi: 10.1158/2159-8290.CD-22-0845 36196574

[147] Ponzo M Debesset A Cossutta M Chalabi-Dchar M Houppe C Pilon C. Nucleolin therapeutic targeting decreases pancreatic cancer immunosuppression. Cancers (Basel). (2022) 14:4265. doi: 10.3390/cancers14174265 36077801 PMC9454580

[148] Kong W Liu Z Sun M Liu H Kong C Ma J. Synergistic autophagy blockade and VDR signaling activation enhance stellate cell reprogramming in pancreatic ductal adenocarcinoma. Cancer Lett. (2022) 539:215718. doi: 10.1016/j.canlet.2022.215718 35526650

[149] Husain K Villalobos-Ayala K Laverde V Vazquez OA Miller B Kazim S. Apigenin targets microRNA-155, enhances SHIP-1 expression, and augments anti-tumor responses in pancreatic cancer. Cancers (Basel). (2022) 14:3613. doi: 10.3390/cancers14153613 35892872 PMC9331563

[150] Fu X Sun G Tu S Fang K Xiong Y Tu Y. Hsa_circ_0046523 mediates an immunosuppressive tumor microenvironment by regulating miR-148a-3p/PD-L1 axis in pancreatic cancer. Front Oncol. (2022) 12:877376. doi: 10.3389/fonc.2022.877376 35712476 PMC9192335

[151] Zhang Z Chen WQ Zhang SQ Bai JX Lau CL Sze SC. The human cathelicidin peptide LL-37 inhibits pancreatic cancer growth by suppressing autophagy and reprogramming of the tumor immune microenvironment. Front Pharmacol. (2022) 13:906625. doi: 10.3389/fphar.2022.906625 35935871 PMC9355328

[152] Cong H Gao J Wang Q Du M Li H Li Q. Increased expression of mitochondrial UQCRC1 in pancreatic cancer impairs antitumor immunity of natural killer cells via elevating extracellular ATP. Front Oncol. (2022) 12:872017. doi: 10.3389/fonc.2022.872017 35769718 PMC9234308

[153] Somani VK Zhang D Dodhiawala PB Lander VE Liu X Kang LI. IRAK4 signaling drives resistance to checkpoint immunotherapy in pancreatic ductal adenocarcinoma. Gastroenterology. (2022) 162:2047–62. doi: 10.1053/j.gastro.2022.02.035 PMC9387774 35271824

[154] Pham TN Spaulding C Shields MA Metropulos AE Shah DN Khalafalla MG. Inhibition of MNKs promotes macrophage immunosuppressive phenotype to limit CD8+ T cell antitumor immunity. JCI Insight. (2022) 7:e152731. doi: 10.1172/jci.insight.152731 35380995 PMC9090262

[155] Wang J Saung MT Li K Fu J Fujiwara K Niu N. CCR2/CCR5 inhibitor permits the radiation-induced effector T cell infiltration in pancreatic adenocarcinoma. J Exp Med. (2022) 219:e20211631. doi: 10.1084/jem.20211631 35404390 PMC9006312

[156] Zhuang H Chen X Wang Y Huang S Chen B Zhang C. Identification of LIPH as an unfavorable biomarkers correlated with immune suppression or evasion in pancreatic cancer based on RNA-seq. Cancer Immunol. (2022) 71:601–12. doi: 10.1007/s00262-021-03019-x PMC10992541 34279685

[157] Ge Y Liu H Zhang Y Liu J Yan R Xiao Z. Inhibition of DCLK1 kinase reverses epithelial-mesenchymal transition and restores T-cell activity in pancreatic ductal adenocarcinoma. Transl Oncol. (2022) 17:101317. doi: 10.1016/j.tranon.2021.101317 34998236 PMC8739467

[158] Brouwer T Ijsselsteijn M Oosting J Ruano D van der Ploeg M Dijk F. A paradoxical role for regulatory T cells in the tumor microenvironment of pancreatic cancer. Cancers (Basel). (2022) 14:3862. doi: 10.3390/cancers14163862 36010856 PMC9405872

[159] Charbonnier LM Wang S Georgiev P Sefik E Chatila TA. Control of peripheral tolerance by regulatory T cell-intrinsic Notch signaling. Nat Immunol. (2015) 16:1162–73. doi: 10.1038/ni.3288 PMC4618075 26437242

[160] Ravi R Noonan KA Pham V Bedi R Zhavoronkov A Ozerov IV. Bifunctional immune checkpoint-targeted antibody-ligand traps that simultaneously disable TGFβ enhance the efficacy of cancer immunotherapy. Nat Commun. (2018) 9:741. doi: 10.1038/s41467-017-02696-6 29467463 PMC5821872

[161] Dawson JC Serrels A Stupack DG Schlaepfer DD Frame MC. Targeting FAK in anticancer combination therapies. Nat Rev Cancer. (2021) 21:313–24. doi: 10.1038/s41568-021-00340-6 PMC8276817 33731845

[162] Li S Mirlekar B Johnson BM Brickey W Wrobel JA Yang N. STING-induced regulatory B cells compromise NK function in cancer immunity. Nature. (2022) 610:373–80. doi: 10.1038/s41586-022-05254-3 PMC9875944 36198789

[163] Zhang X Lao M Xu J Duan Y Yang H Li M. Combination cancer immunotherapy targeting TNFR2 and PD-1/PD-L1 signaling reduces immunosuppressive effects in the microenvironment of pancreatic tumors. J Immunother Cancer. (2022) 10:e003982. doi: 10.1136/jitc-2021-003982 35260434 PMC8906048

[164] Liu X Liu J Xu J Zhang B Wei M Li J. Intra-tumoral infiltration of adipocyte facilitates the activation of antitumor immune response in pancreatic ductal adenocarcinoma. Transl Oncol. (2023) 27:101561. doi: 10.1016/j.tranon.2022.101561 36257208 PMC9576545

[165] Daßler-Plenker J Küttner V Egeblad M. Communication in tiny packages: Exosomes as means of tumor-stroma communication. Biochim Biophys Acta Rev Cancer. (2020) 1873:188340. doi: 10.1016/j.bbcan.2020.188340 31926290

[166] Oliveira C Calmeiro J Carrascal MA Falcão A Gomes C Miguel Neves B. Exosomes as new therapeutic vectors for pancreatic cancer treatment. Eur J Pharm Biopharm. (2021) 161:4–14. doi: 10.1016/j.ejpb.2021.02.002 33561524

[167] Leinwand J Miller G. Regulation and modulation of antitumor immunity in pancreatic cancer. Nat Immunol. (2020) 21:1152–9. doi: 10.1038/s41590-020-0761-y 32807942

[168] Zaghdoudi S Decaup E Belhabib I Samain R Cassant-Sourdy S Rochotte J. FAK activity in cancer-associated fibroblasts is a prognostic marker and a druggable key metastatic player in pancreatic cancer. EMBO Mol Med. (2020) 12:e12010. doi: 10.15252/emmm.202012010 33025708 PMC7645544

[169] Jiang H Liu X Knolhoff BL Hegde S Lee KB Jiang H. Development of resistance to FAK inhibition in pancreatic cancer is linked to stromal depletion. Gut. (2020) 69:122–32. doi: 10.1136/gutjnl-2018-317424 PMC7167297 31076405

[170] Yauch RL Gould SE Scales SJ Tang T Tian H Ahn CP. A paracrine requirement for hedgehog signalling in cancer. Nature. (2008) 455:406–10. doi: 10.1038/nature07275 18754008

[171] Shang S Hua F Hu Z-W. The regulation of β-catenin activity and function in cancer: therapeutic opportunities. Oncotarget. (2017) 8:33972–89. doi: 10.18632/oncotarget.15687 PMC5464927 28430641

[172] Spranger S Bao R Gajewski TF. Melanoma-intrinsic β-catenin signalling prevents anti-tumour immunity. Nature. (2015) 523:231–5. doi: 10.1038/nature14404 25970248

[173] Du W Menjivar RE Donahue KL Kadiyala P Velez-Delgado A Brown KL. WNT signaling in the tumor microenvironment promotes immunosuppression in murine pancreatic cancer. J Exp Med. (2023) 220:e20220503. doi: 10.1084/jem.20220503 36239683 PMC9577101

[174] Ansari D Ohlsson H Althini C Bauden M Zhou Q Hu D. The Hippo signaling pathway in pancreatic cancer. Anticancer Res. (2019) 39:3317–21. doi: 10.21873/anticanres.13474 31262852

[175] Kumar S Schoonderwoerd MJA Kroonen JS de Graaf IJ Sluijter M Ruano D. Targeting pancreatic cancer by TAK-981: a SUMOylation inhibitor that activates the immune system and blocks cancer cell cycle progression in a preclinical model. Gut. (2022) 71:2266–83. doi: 10.1136/gutjnl-2021-324834 PMC9554032 35074907

[176] Sarhan D Eisinger S He F Bergsland M Pelicano C Driescher C. Targeting myeloid suppressive cells revives cytotoxic anti-tumor responses in pancreatic cancer. iScience. (2022) 25:105317. doi: 10.1016/j.isci.2022.105317 36310582 PMC9615326

[177] He S Gu X Yang J Xu F Hu J Wang W. Sphingomyelin synthase 2 is a positive regulator of the CSF1R-STAT3 pathway in pancreatic cancer-associated macrophage. Front Pharmacol. (2022) 13:902016. doi: 10.3389/fphar.2022.902016 36324684 PMC9618885

[178] Li H Zhu R Liu X Zhao K Hong D. Siglec-15 regulates the inflammatory response and polarization of tumor-associated macrophages in pancreatic cancer by inhibiting the cGAS-STING signaling pathway. Oxid Med Cell Longev. (2022) 2022:3341038. doi: 10.1155/2022/3341038 36105484 PMC9467737

[179] Sim W Lim WM Hii LW Leong CO Mai CW. Targeting pancreatic cancer immune evasion by inhibiting histone deacetylases. World J Gastroenterol. (2022) 28:1934–45. doi: 10.3748/wjg.v28.i18.1934 PMC9150054 35664961

[180] Ibrahim AM Wang YH. Viro-immune therapy: A new strategy for treatment of pancreatic cancer. World J Gastroenterol. (2016) 22:748–63. doi: 10.3748/wjg.v22.i2.748 PMC4716074 26811622

[181] Hopkins AC Yarchoan M Durham JN Yusko EC Rytlewski JA Robins HS. T cell receptor repertoire features associated with survival in immunotherapy-treated pancreatic ductal adenocarcinoma. JCI Insight. (2018) 3:e122092. doi: 10.1172/jci.insight.122092 29997287 PMC6124515

[182] Suarez-Carmona M Williams A Schreiber J Hohmann N Pruefer U Krauss J. Combined inhibition of CXCL12 and PD-1 in MSS colorectal and pancreatic cancer: modulation of the microenvironment and clinical effects. J Immunother Cancer. (2021) 9:e002505. doi: 10.1136/jitc-2021-002505 34607895 PMC8491418

[183] Wang X Li X Wei X Jiang H Lan C Yang S. PD-L1 is a direct target of cancer-FOXP3 in pancreatic ductal adenocarcinoma (PDAC), and combined immunotherapy with antibodies against PD-L1 and CCL5 is effective in the treatment of PDAC. Signal Transduct Target Ther. (2020) 5:38. doi: 10.1038/s41392-020-0144-8 32300119 PMC7162990

[184] Overman M Javle M Davis RE Vats P Kumar-Sinha C Xiao L. Randomized phase II study of the Bruton tyrosine kinase inhibitor acalabrutinib, alone or with pembrolizumab in patients with advanced pancreatic cancer. J Immunother Cancer. (2020) 8:e000587. doi: 10.1136/jitc-2020-000587 32114502 PMC7057435

[185] Buoncervello M Gabriele L Toschi E. The janus face of tumor microenvironment targeted by immunotherapy. Int J Mol Sci. (2019) 20:4320. doi: 10.3390/ijms20174320 31484464 PMC6747403

[186] McGranahan N Furness AJ Rosenthal R Ramskov S Lyngaa R Saini SK. Clonal neoantigens elicit T cell immunoreactivity and sensitivity to immune checkpoint blockade. Science. (2016) 351:1463–9. doi: 10.1126/science.aaf1490 PMC4984254 26940869

[187] Saxena M Bhardwaj N. Re-emergence of dendritic cell vaccines for cancer treatment. Trends Cancer. (2018) 4:119–37. doi: 10.1016/j.trecan.2017.12.007 PMC5823288 29458962

[188] Mougel A Terme M Tanchot C. Therapeutic cancer vaccine and combinations with antiangiogenic therapies and immune checkpoint blockade. Front Immunol. (2019) 10:467. doi: 10.3389/fimmu.2019.00467 30923527 PMC6426771

[189] Yin C Alqahtani A Noel MS. The next frontier in pancreatic cancer: targeting the tumor immune milieu and molecular pathways. Cancers (Basel). (2022) 14:2619. doi: 10.3390/cancers14112619 35681599 PMC9179513

[190] Schmidts A Maus MV. Making CAR T cells a solid option for solid tumors. Front Immunol. (2018) 9:2593. doi: 10.3389/fimmu.2018.02593 30467505 PMC6235951

[191] Schuster SJ Svoboda J Chong EA Nasta SD Mato AR Anak Ö. Chimeric antigen receptor T cells in refractory B-cell lymphomas. N Engl J Med. (2017) 377:2545–54. doi: 10.1056/NEJMoa1708566 PMC5788566 29226764

[192] Wang X Chang WC Wong CW Colcher D Sherman M Ostberg JR. A transgene-encoded cell surface polypeptide for selection, in *vivo* tracking, and ablation of engineered cells. Blood. (2011) 118:1255–63. doi: 10.1182/blood-2011-02-337360 PMC3152493 21653320

[193] Ali AI Wang M von Scheidt B Dominguez PM Harrison AJ Tantalo DGM. A histone deacetylase inhibitor, panobinostat, enhances chimeric antigen receptor T-cell antitumor effect against pancreatic cancer. Clin Cancer Res. (2021) 27:6222–34. doi: 10.1158/1078-0432.CCR-21-1141 34475103

[194] Da Y Liu Y Hu Y Liu W Ma J Lu N. STING agonist cGAMP enhances antitumor activity of CAR-NK cells against pancreatic cancer. Oncoimmunology. (2022) 11:2054105. doi: 10.1080/2162402X.2022.2054105 35371622 PMC8967397

[195] Leidner R Sanjuan Silva N Huang H Sprott D Zheng C Shih YP. Neoantigen T-cell receptor gene therapy in pancreatic cancer. N Engl J Med. (2022) 386:2112–9. doi: 10.1056/NEJMoa2119662 PMC9531755 35648703

[196] Byrne KT Betts CB Mick R Sivagnanam S Bajor DL Laheru DA. Neoadjuvant selicrelumab, an agonist CD40 antibody, induces changes in the tumor microenvironment in patients with resectable pancreatic cancer. Clin Cancer Res. (2021) 27:4574–86. doi: 10.1158/1078-0432.CCR-21-1047 PMC8667686 34112709

[197] Byrne KT Vonderheide RH. CD40 stimulation obviates innate sensors and drives T cell immunity in cancer. Cell Rep. (2016) 15:2719–32. doi: 10.1016/j.celrep.2016.05.058 PMC4917417 27292635

[198] Zhang H Ye L Yu X Jin K Wu W. Neoadjuvant therapy alters the immune microenvironment in pancreatic cancer. Front Immunol. (2022) 13:956984. doi: 10.3389/fimmu.2022.956984 36225934 PMC9548645

